# Supplementary material for: Incidence and prognostic factors of self-harm and subsequent unnatural death in South Africa: A cohort study
Source: PLoS Med. 2025 Sep 26;22(9):e1004765. doi: 10.1371/journal.pmed.1004765 (PMC12503312; doi:10.1371/journal.pmed.1004765)
Supplement: S1 Strobe Checklist — https://www.strobe-statement.org/. (PDF) [file pmed.1004765.s001.pdf]

STROBE Statement—Checklist of items that should be included in reports of ***cohort studies***

	Item No	Recommendation	Section, subheading or paragraph number
**Title and abstract**	1	(*a*) Indicate the study's design with a commonly used term in the title or the abstract	Title
		(*b*) Provide in the abstract an informative and balanced summary of what was done and what was found	Abstract
**Introduction**			
Background/rationale	2	Explain the scientific background and rationale for the investigation being reported	Introduction, paragraph 5 and 6
Objectives	3	State specific objectives, including any prespecified hypotheses	Introduction, paragraph 6
**Methods**			
Study design	4	Present key elements of study design early in the paper	Methods, paragraph 1
Setting	5	Describe the setting, locations, and relevant dates, including periods of recruitment, exposure, follow-up, and data collection	Methods, Setting
Participants	6	(*a*) Give the eligibility criteria, and the sources and methods of selection of participants. Describe methods of follow-up	Methods, Study design and participants, Figure 1
		(*b*) For matched studies, give matching criteria and number of exposed and unexposed	Methods, Statistical analysis, Post-hoc analysis
Variables	7	Clearly define all outcomes, exposures, predictors, potential confounders, and effect modifiers. Give diagnostic criteria, if applicable	Methods, outcomes, predictors
Data sources/ measurement	8*	For each variable of interest, give sources of data and details of methods of assessment (measurement). Describe comparability of assessment methods if there is more than one group	Methods, Data sources
Bias	9	Describe any efforts to address potential sources of bias	Methods, Statistical analysis, Text B and C in Appendix S4
Study size	10	Explain how the study size was arrived at	Figure 1
Quantitative variables	11	Explain how quantitative variables were handled in the analyses. If applicable, describe which groupings were chosen and why	Methods, Predictors
Statistical methods	12	(*a*) Describe all statistical methods, including those used to control for confounding	Methods, Statistical analysis
		(*b*) Describe any methods used to examine subgroups and interactions	Methods, Statistical analysis
		(*c*) Explain how missing data were addressed	NA, individuals with missing sex, age, or death linkage were excluded
		(*d*) If applicable, explain how loss to follow-up was addressed	NA

		(*e*) Describe any sensitivity analyses	Methods, Sensitivity analysis
**Results**			
Participants	13*	(a) Report numbers of individuals at each stage of study—eg numbers potentially eligible, examined for eligibility, confirmed eligible, included in the study, completing follow-up, and analysed<br>(b) Give reasons for non-participation at each stage<br>(c) Consider use of a flow diagram	Figure 1<br>NA<br>Figure 1
Descriptive data	14*	(a) Give characteristics of study participants (eg demographic, clinical, social) and information on exposures and potential confounders<br>(b) Indicate number of participants with missing data for each variable of interest<br>(c) Summarise follow-up time (eg, average and total amount)	Results, Characteristics of study population, Table 1 and Table 2<br>Unknown death shown in Table 1. Few individuals with missing sex or age were excluded (see Figure 1).<br>Results, Characteristics of study population, Time at risk shown in Table A in Appendix S4
Outcome data	15*	Report numbers of outcome events or summary measures over time	Table A in Appendix S4 for self-harm events and Results, Mortality after non-fatal self-harm and Table 2 for mortality

Main results	16	(*a*) Give unadjusted estimates and, if applicable, confounder-adjusted estimates and their precision (eg, 95% confidence interval). Make clear which confounders were adjusted for and why they were included<br><br>(*b*) Report category boundaries when continuous variables were categorized<br><br>(*c*) If relevant, consider translating estimates of relative risk into absolute risk for a meaningful time period	Completely unadjusted estimates are not informative due to strong confounding by age and sex. Age and sex adjusted results reported in addition to fully adjusted models in Figure 3 and Figure 5. Methods, Predictors Incidence and cumulative incidence are reported in addition to hazard ratios
Other analyses	17	Report other analyses done—eg analyses of subgroups and interactions, and sensitivity analyses	Text A-C in Appendix S4
**Discussion**			
Key results	18	Summarise key results with reference to study objectives	Discussion, paragraph 1
Limitations	19	Discuss limitations of the study, taking into account sources of potential bias or imprecision. Discuss both direction and magnitude of any potential bias	Discussion, paragraph 7
Interpretation	20	Give a cautious overall interpretation of results considering objectives, limitations, multiplicity of analyses, results from similar studies, and other relevant evidence	Discussion, paragraph 2-6
Generalisability	21	Discuss the generalisability (external validity) of the study results	Discussion, paragraph 6
**Other information**			
Funding	22	Give the source of funding and the role of the funders for the present study and, if applicable, for the original study on which the present article is based	Declarations Role of the funding source

*Give information separately for exposed and unexposed groups.

Note: An Explanation and Elaboration article discusses each checklist item and gives methodological background and published examples of transparent reporting. The STROBE checklist is best used in conjunction with this article (freely available on the Web sites of PLoS Medicine at http://www.plosmedicine.org/, Annals of Internal Medicine at http://www.annals.org/, and Epidemiology at http://www.epidem.com/). Information on the STROBE Initiative is available at http://www.strobe-statement.org.
