# Supplementary material for: Incidence and prognostic factors of self-harm and subsequent unnatural death in South Africa: A cohort study
Source: PLoS Med. 2025 Sep 26;22(9):e1004765. doi: 10.1371/journal.pmed.1004765 (PMC12503312; doi:10.1371/journal.pmed.1004765)
Supplement: S1 Appendix — (PDF) [file pmed.1004765.s002.pdf]

CONCEPT SHEET: REGIONAL ANALYSES

**Steering Group approval date:**	*NA*
**Tracking number:**	SA318
**Title:**	Health care contacts for intentional self-harm and subsequent mortality from unnatural causes among people living with and without HIV: a cohort study of beneficiaries of a South African medical insurance scheme
**Lead author:**<br>**Email:**	Veronika Whitesell-Skrivankova
**IeDEA senior investigator:**<br>**Email:**	Andreas Haas<br><br>andreas.haas@unibe.ch
**Type of c0ncept**	*Select as appropriate:*<br>☒ New concept – no linked conference abstract<br>☐ New concept – linked to conference abstract which **has not been** approved by SG<br>☐ New concept – linked to conference abstract which **has been** approved by SG<br>☐ Existing concept – major revisions requiring SG approval
**Type of study**	*Select as appropriate:*<br>☐ Full research study – multiple sites<br>☒ Full research study – single site<br>☐ Study protocol<br>☐ Fast track study using existing dataset<br>☐ Mathematical or methodological modelling *(indicate if this will use IeDEA-SA data)*<br>☐ Systematic review
**Statisticians:**<br>**Email:**	Veronika Whitesell-Skrivankova
**Data manager:**<br>**Email:**	Chido Chinogurei, Andreas Haas

**Where will statistical analyses be done?**	ISPM
**Required variables:**	Concept will be using AfA and AfA controls data. The following variables will be used:<br>• Demographics characteristics: age, and sex<br>• Start and end of insurance coverage<br>• Outpatient and hospital claims for intentional self-harm and events of undetermined event<br>• Mental health diagnoses<br>• HIV status<br>• Vital registration data from the National Population Register including cause of death information: natural/unnatural/unknown causes
**Target journal:**	
**Ethics:**	*Select as appropriate:*<br>☒ This concept uses only the IeDEA-SA standard dataset and is covered by the core IeDEA-SA ethics approvals.<br>☐ This concept requires additional collection of health-related data, measurements or tests, or sampling of biological material not included in the IeDEA-SA standard dataset. Additional ethics approval is required.* (Describe ethical considerations for any additional data collection here, including responsible IRBs.)
**Milestones:**	*Circulation of concept sheet:* *5. September 2023*<br>*Ethics approval (for additional data collection):* *NA*<br>*Circulation of mature draft paper:* *<date>*<br>*Submission to target journal:* *<date>*
**Abstract:**<br>(about 100 words)	Background: Individuals with a history of suicide attempts or specific mental disorders are at a very high risk for suicide. Despite available data on these predictors, they remain underutilized in identifying high-risk individuals for targeted interventions.<br><br>Objectives: This study aims to examine the incidence and risk factors of intentional self-harm events, reattempts, and subsequent mortality from unnatural causes among beneficiaries of a private medical insurance scheme.<br><br>Methods: Cohort study using national vital registration and reimbursement claims data from beneficiaries of a South African medical insurance scheme and an HIV management program, aged 10 years or older, to analyze the risks and patterns of intentional and potential self-harm events from 2011 to 2022. Survival methods, Poisson regression, and machine learning algorithms will be used for analysis.

* If additional ethics approvals are required, a copy must be sent to the ISPM Program Manager before data collection can begin.

Background

Suicide is a major public health concern. Globally, every year approximately 700,000 people die by suicide, making it a leading cause of death for younger age groups.[1] Most suicides (77%) occur in low-and-middle-income countries.[1] In South Africa, 13,774 suicides were recorded in 2019, placing it among the ten countries with the highest suicide rates globally.[1] Almost 80% of these suicides occurred in males.[1] Suicide attempts are far more common than completed suicides. In South Africa, the lifetime prevalence of suicide attempts is 2.9% overall, with variations among different demographic groups: 1.8% in males, 3.8% in females, 2.4% among Black Africans, 7.1% among those of Mixed Ancestry, 2.4% among Whites, and 2.5% among Indians.[2] Young adults aged 18-34 years are at an almost 12-fold risk of suicide attempts compared to adults 65 years or older.[2] The lifetime prevalence of suicidal ideation is estimated at 8.0% for males and 10.1% for females.[2]

Suicide risk factors are multifaceted, ranging from the individual to relationship, community, and societal levels.[3] The by far strongest risk factor of suicide attempts and suicide is a history of a previous suicide attempt. Repeated suicide attempts and completed suicides are common among individuals who survived a first attempt.[4, 5] A meta-analysis of 103 studies estimated a rate of repeated attempts of 16.3% at 1 year, 21.4% at 2 years, and 23.2% at 3 years after the index attempt.[4] Another meta-analysis of 41 studies estimated a suicide mortality rate of 2.8% at 1 year, 5.6% at 5 years, and 7.4% at 10 years after a nonfatal suicide attempt.[5] In comparison to the estimated global suicide rate of 9.4 suicides per 100,000 person-years,[6] the suicide mortality rate after an index attempt is increased by approximately 300 at 1 year, 120 at 5 years, and 80 at 10 years after the index attempt.[4] The suicide method used in a nonfatal index attempt is a strong predictor of subsequent suicide mortality.[7] Most individuals who died by suicide following a repeated attempt used the same method as in their initial attempt. Consequently, individuals who employed a more lethal methods during their index attempt are at a higher risk of subsequent suicide mortality. The highest suicide rates are observed among individuals who attempted suicide by hanging, strangulation, or suffocation. This is followed by those who attempted suicide through gassing, jumping from a height, using firearms or explosives, or drowning. Notably, lower suicide mortality rates were observed among individuals who survived attempts using less lethal methods, such as cutting and poisoning.[7]

The second most significant risk factor for suicide is mental illness. A meta-analysis of data from over 15,000 suicides across more than 50 countries revealed that 97% of individuals who died by suicide had been diagnosed with a psychiatric disorder. The most prevalent psychiatric disorders were mood disorders (36%), including bipolar disorder and depression, followed by substance use disorders (22%), personality disorders (12%), and schizophrenia (11%).[8] In a systematic review of 20 studies, major depressive disorder was associated with the highest increase in suicide risk, at nearly an 8-fold increase, compared to individuals without psychiatric disorders. This was followed by bipolar disorders and schizophrenia with a 6-fold increase, anxiety disorders with a 5-fold increase, and dysthymia with a 4-fold increase.[9]

The risk of suicide is also associated with socioeconomic factors and other individual, relationship, community, and societal levels factors. Risk of suicide is approximately doubled for individuals with low income compared to those with high income, for manual, non-skilled, and blue-collar workers compared to highly qualified individuals, for individuals affected by unemployment compared to the employed, and for those with less than secondary education compared to those with higher education.[10] Additional individual-level risk factors for suicide include alcohol abuse, financial crises, feelings of hopelessness, and chronic pain. Furthermore, a family history of suicide, as well as genetic and biological factors, can increase the risk of suicide.[3] On the relationship level, persons who are single, divorced, or widowed often face heightened risks.[3] A pronounced sense of isolation, the absence of a supportive social network, persistent relationship conflicts, and recent losses can exacerbate these vulnerabilities.[3] At the community level, experiences such as exposure to disasters, wars, conflicts, dislocations, discrimination, trauma, and abuse can significantly intensify the risk of

suicide.[3] On a societal level, factors such as easy access to lethal means and inappropriate media reporting on suicides, increasing the risk of 'copycat' suicides, can inadvertently contribute to its prevalence.[3]

Despite the availability of data from insurance claims on the strongest predictors of suicide mortality, specifically previous attempts and mental disorders, these data are not currently used to identify high-risk individuals who could benefit from targeted suicide prevention interventions. This study aims to examine the incidence and risk factors of intentional self-harm events, reattempts, and subsequent mortality from unnatural causes among beneficiaries of a private medical insurance scheme. Results may inform the development of an intervention that leverages available data sources to identify individuals at the highest risk of suicide and suicide attempts, and then provide targeted support to reduce this risk.

Objectives

1. Estimate the incidence of intentional self-harm events and potential self-harm events with undetermined intent.
2. Estimate the incidence of repeated intentional or potential self-harm events following an initial event.
3. Investigate the risk factors associated with intentional or potential self-harm events.
4. Examine the incidence of mortality from unnatural causes among individuals with intentional, potential, or no history of self-harm events.
5. Evaluate intentional or potential self-harm events, mental disorder, sociodemographic characteristics as predictors of mortality from unnatural causes.
6. Build a predictive model to forecast mortality from unnatural causes among individuals with a previous intentional or potential self-harm event.

Study design and participants

We will analyse national vital registration data along with longitudinal inpatient and outpatient reimbursement claims data from a South African general medical insurance scheme and a South African HIV disease management program. We will include adolescents and adults aged 10 years or older who were covered by the medical insurance scheme at any time between 1 January 2011 and 30 June 2020, or those enrolled in the HIV disease management program at any time between 1 January 2011 to 31 October 2022. Individuals with age will be excluded from the analysis. Those with an unconfirmed vital status who could not be linked to the vital registration system, will be excluded from the mortality analysis. To prevent duplication, members of the general medical insurance scheme who have enrolled with AfA will be excluded from the general medical insurance scheme dataset.

Measures

Intentional or potential self-harm events

We are considering intentional self-harm (ICD10 codes X60-X84) and potential self-harm events of undetermined intent (Y10-Y29), where available information is insufficient to enable a medical or legal authority to make a distinction between accident, self-harm and assault.[11] Intentional self-harm events are categorized by method, including: firearms or explosives (X72-X75); drowning and submersion (X71); hanging, strangulation, and suffocation (X70); self-poisoning by exposure to gases (X67); jumping from a high place or in front of a moving object (X80-X81); smoke, fire, and flames (X76); crashing of a motor vehicle (X82); exposure to steam, hot vapours, and hot objects (X77); self-poisoning methods other than gas (X60-X66 and X68-X69); injuries from sharp objects (X78); blunt objects (X79); and other or unspecified means (X83-X84). For events of undetermined intent, the classifications include: firearms or explosives (Y22-Y25); drowning and submersion (Y21); hanging, strangulation, and suffocation (Y20); poisoning by gases (Y17); jumping from a height or before a moving object (Y30-Y31); smoke, fire, and flames (Y26); vehicular crashes (Y32); steam, hot vapours,

and hot objects (Y27); poisoning excluding gases (Y10-Y16 and Y18-Y19); sharp objects (Y28); blunt objects (Y29). We did not consider other or unspecified events of undetermined intent (Y33-Y34).

Identifying repeated self-harm events based on claims data is challenging because claims with relevant ICD-10 codes submitted after an initial event might either indicate a repeated event or follow-up care for the initial event. To minimize the risk of misclassifying follow-up care as a new event, we considered self-harm claims submitted within various time windows (7, 14, 30, 60, 90, 180, and 365 days) of a preceding self-harm claim as treatment episode related to the initial event. The initial event of an episode was determined based on the data from all claims linked to that episode, prioritizing intentional self-harm over potential self-harm, more lethal methods over less lethal ones, and hospital treatments over outpatient care. We will implement three sets of definitions to identify repeated self-harm events. 1) Considering only intentional self-harm events (high certainty), 2) considering both intentional self-harm events and potential self-harm events of undetermined intent (low certainty), and 3) considering potential self-harm events of undetermined intent only in individuals with evidence of a previous intentional self-harm event (moderate certainty).

Mental disorders

Mental disorders will be classified according to ICD-10 diagnosis and grouped as organic mental disorders (F00-09), substance use disorder (F10-F16, F18-F19), psychotic disorder (F20-29), bipolar disorder (F31), depression (F32, F33, F34.1), anxiety disorder (F40-48), behavioural syndrome associated with physical factors (F50-F59), personality disorder (F60-F69), intellectual disabilities (F70-F79), developmental disorder (F80-F89), or a behavioural disorder (F90-F98).

HIV status

We will consider individuals with one of the following HIV indicators as living with HIV: HIV-related ICD-10 diagnoses (B20-24, F02.4, O98.7, R75, Z21), HIV-related laboratory tests (positive HIV test, HIV RNA viral load measurement, CD4 cell count measurement), ATC codes for antiretroviral therapy (ART) or registration in the Aid for AIDS (AfA) disease management program.

Sociodemographic measures

We will consider the following sociodemographic characteristics: age (10-19, 20-29, 30-39, 40-49, 50-59, 60-69, and ≥70 years), sex (male, female, other/unknown).

Mortality

We will determine the vital status of beneficiaries using mortality records from both the vital registration system and the medical insurance database. If there are discrepancies in the death dates between the sources, we will prioritise the dates provided by the vital registration system. Causes of death will be categorized into natural, unnatural, or unknown. Unnatural causes include all deaths resulting from external factors, such as suicides, homicides, accidents, medical errors, alcohol intoxications, and drug overdoses (ICD10 V01—Y98). Natural causes include all deaths from chapters 1 to 18 of the ICD-10, as outlined by Statistics South Africa. Unknown causes will include deaths that are under investigation at the time of linkage, deaths due to unidentified causes, and deaths recorded only by the medical insurance.

Statistical analysis

We will conduct descriptive analyses to assess the sociodemographic characteristics of the beneficiaries and prevalence of mental disorders by sex. In all longitudinal analysis, HIV status, mental disorders, and history of prior self-harm will be modelled as time updated variables.

Objective 1: First, we will compute crude incidence rates for first intentional and potential self-harm events per 100,000 person-years, stratified by sex and HIV status, by dividing the number of individuals with an incident event by the total person-years at risk. Second, we will estimate the cumulative incidence of a first intentional and potential self-harm event by sex, HIV status. Third, we will use

Royston-Parmar flexible parametric survival models to assess the absolute risk of intentional and potential self-harm. We will estimate incidence rates per 100,000 person-years with 95% confidence intervals (CIs) as a continuous function of age, stratified by sex and HIV status. We will consider one to eight degrees of freedom for the natural spline basis for the incidence rate and one to six degrees of freedom for the natural splines modelling an interaction between HIV status, sex, and age.

Objective 2: We will compute crude incidence rates and estimate the cumulative incidence of repeated intentional and potential self-harm events, stratified by sex and HIV status. For this analysis, beneficiaries will be tracked from the date of their first event.

Objective 3: We will estimate the adjusted hazard ratios (aHR) with 95% CIs for risk factors associated with a first intentional and potential self-harm event using Cox proportional hazards models. Risk factors in this model will include sociodemographic characteristics, mental disorders, and HIV status. For the analysis of repeated events, we will compute rate ratios using Poisson offset models, considering the same set of risk factors along with prior self-harm events.

Objective 4: Using the Aalen-Johansen estimator, we will estimate the cumulative incidence of death from unnatural causes after an intentional, potential, or repeated self-harm event, in comparison to those with no self-harm history, considering other causes of death as competing events. Analysis will be stratified by sex and HIV status.

Objective 5: We will estimate cause-specific aHR with 95% CIs for risk factors of death from unnatural causes using Cox proportional hazards models. Included risk factors in this model will be sociodemographic characteristics, mental disorders, HIV status, and previous self-harm events and method. We will incorporate relevant two-way interactions in the model. To provide clear interpretations, we will estimate the marginal hazard ratios for each risk factor, averaging over the distribution of interacting variables.

Objective 6: Informed by domain knowledge, results from objective 5, and machine learning feature selection techniques we will select relevant predictions. We will use survival analysis techniques and machine learning algorithms such as random survival forests, gradient boosted Cox regression, and Cox proportional hazards regression to construct the predictive model base on the training dataset. Using the test dataset, we will validate the performance of the model in predicting the risk of mortality from unnatural causes. Metrics like Area Under the Curve (AUC), sensitivity, specificity, and overall accuracy will be employed to assess the model's effectiveness. Based on the predicted survival probabilities from our model, we will categorize individuals into different risk groups (e.g., low, medium, high risk) for mortality to better guide clinical interventions. We will employ techniques such as SHAP (SHapley Additive exPlanations) values or feature importance plots to interpret the model, providing insights into which factors are most influential in predicting the risk of mortality.

References

1 World Health Organization. Suicide worldwide in 2019: global health estimate. 2021.

2 Joe S, Stein DJ, Seedat S, Herman A, Williams DR. Non-fatal suicidal behavior among South Africans. *Soc Psychiatry Psychiatr Epidemiol* 2008; **43**: 454–61.

3 WHO., Saxena S, Saxena S, *et al.* Preventing Suicide : a Global Imperative. World Health Organization, 2014.

4 de la Torre-Luque A, Pemau A, Ayad-Ahmed W, *et al.* Risk of suicide attempt repetition after an index attempt: A systematic review and meta-analysis. *Gen Hosp Psychiatry* 2023; **81**: 51–6.

5 Demesmaeker A, Chazard E, Hoang A, Vaiva G, Amad A. Suicide mortality after a nonfatal suicide attempt: A systematic review and meta-analysis. *Aust N Z J Psychiatry* 2022; **56**: 603–16.

6 Institute for Health Metrics and Evaluation. GHDX - GBD Results Tool. Institute for Health Metrics and Evaluation. 2021; : 4–5.

7 Runeson B, Tidemalm D, Dahlin M, Lichtenstein P, Langstrom N. Method of attempted suicide as predictor of subsequent successful suicide: national long term cohort study. *BMJ* 2010; **341**: c3222–c3222.

8 Bertolote J, Fleischmann A. Suicide and psychiatric diagnosis: a worldwide perspective . *World Psychiatry* 2002; **1**: 181–5.

9 Moitra M, Santomauro D, Degenhardt L, *et al.* Estimating the risk of suicide associated with mental disorders: A systematic review and meta-regression analysis. *J Psychiatr Res* 2021; **137**: 242–9.

10 Li Z, Page A, Martin G, Taylor R. Attributable risk of psychiatric and socio-economic factors for suicide from individual-level, population-based studies: A systematic review. *Soc Sci Med* 2011; **72**: 608–16.

11 World Health Organization (WHO). International Statistical Classification of Diseases and Related Health Problems 10th Revision. 2016. https://icd.who.int/browse10/2016/en (accessed Aug 25, 2019).
