# Supplementary material for: Incidence and prognostic factors of self-harm and subsequent unnatural death in South Africa: A cohort study
Source: PLoS Med. 2025 Sep 26;22(9):e1004765. doi: 10.1371/journal.pmed.1004765 (PMC12503312; doi:10.1371/journal.pmed.1004765)
Supplement: S2 Appendix — (PDF) [file pmed.1004765.s003.pdf]

Self-Harm and Unnatural Death in South Africa: A Cohort Study

Table A: Protocol deviations

Protocol section / Analysis	Planned approach	Approach used	Research for deviation
Exposure classification	Analyze intentional self-harm (ISH, ICD10 codes X60-X84) and potential self-harm events of undetermined intent (Y10-Y29) as exposure variables	Analyzed only ISH events	Upon thorough investigation of coding practices and healthcare utilization patterns following events of undetermined intent (ICD-10 codes Y10–Y29), we found that the specificity of these events for intentional self-harm was very low. Although some events of undetermined intent may represent ISH encounters, the majority appear to reflect accidental injuries. Including events of undetermined intent would likely lead to substantial overestimation of self-harm incidence. We therefore restricted our analysis to events explicitly coded as ISH (X60–X84) to ensure a high specificity of our case definition and to align our study with the international literature (e.g. Global Burden of Diseases study) that Is only using ISH codes.
Stratification by HIV status	Stratify most analyses by sex and HIV status	Primary analyses were stratified by sex and age group instead of sex and HIV status. Analyses stratified by sex and HIV status are presented in supplementary materials.	HIV status was only weakly associated with the incidence of self-harm. In contrast, age emerged as a much stronger and more clinically relevant predictor. We therefore chose to stratify primary analyses by age and sex, rather than by sex and HIV status. Incidence by sex, age and HIV status is presented in the supplementary material.
Age categories	Categorize age as 10-19, 20-29, 30-39, 40-49, 50-59, 60-69, and ≥70 years	Categorized age as 10–14, 15–24, 25–39, and ≥40 years.	Original age groups were too granular for visual presentation. We regrouped into broader, more clinically meaningful categories: children (10–14), adolescents and young adults (15–24), adults (25–39), and older adults (≥40).
Mental disorders classification	Classify diagnoses into the following categories: organic mental disorders (F00–F09), substance use disorder (F10–F16, F18–F19), psychotic disorder (F20–F29), bipolar disorder (F31), depression (F32, F33, F34.1), anxiety disorder (F40–F48),	Combined behavioral syndromes (F50–F59), intellectual disabilities (F70–F79), developmental disorders (F80–F89), and behavioral disorders (F90–F98) into a single “other mental disorders” category.	The F50–F59 category is heterogeneous (e.g., eating disorders, sexual dysfunction), making interpretation challenging. The remaining categories had small sample sizes. Grouping these categories avoided presenting the

	behavioral syndrome associated with physical factors (F50–F59), personality disorder (F60–F69), intellectual disabilities (F70–F79), developmental disorders (F80–F89), and behavioral disorders (F90–F98).		behavioral syndromes groups that is not clinically meaningful to readers and ensured sufficient numbers for analysis.
Recurrent episodes	Examine the incidence of repeated ISH events and associated factors	Repeated events were not analyzed	It was not feasible to reliably distinguish between claims submitted for follow-up care after an index ISH event and claims for new ISH episodes. The planned time-based separation of events was not reliable, as many recurrent events occurred soon after the index event, overlapping with the expected follow-up care period.
Predictive model (Objective 6)	Develop a predictive model for unnatural death following intentional self-harm	This analysis was not included in the current manuscript. Results will be reported separately. Manuscript currently in preparation.	Due to the scope and complexity of the predictive modeling, we decided to present these findings in a separate manuscript to allow for a more detailed and focused analysis and presentation.
