# Supplementary material for: Incidence and prognostic factors of self-harm and subsequent unnatural death in South Africa: A cohort study
Source: PLoS Med. 2025 Sep 26;22(9):e1004765. doi: 10.1371/journal.pmed.1004765 (PMC12503312; doi:10.1371/journal.pmed.1004765)
Supplement: S3 Appendix — Table A. Incidence rates of self-harm encounters per 100,000 person-years, stratified by sex, for different age groups and overall. Table B. Cumulative incidence (%) and 95% confidence intervals of self-harm, stratified by sex, for different age groups and overall. Table C. Healthcare utilization before self-harm events compared with matched controls prior to hypothetical matched event dates. Table D. Cumulative incidence (%) and 95% confidence intervals of unnatural death, stratified by sex and self-harm method, and overall. Table E. Characteristics of the study population by cohort. Table F. Characteristics of individuals included and excluded from the mortality analysis. Fig A. Incidence of self-harm by age, sex, and HIV status. Incidence per 100,000 person-years in general population as a continuous function of age, stratified by sex and HIV status. Individuals were censored at the time of their first documented self-harm event. Repeated self-harm events were not considered. Fig B. Time-varying hazard ratios for unnatural death by sex, comparing individuals with and without prior non-fatal self-harm encounter. Hazard ratios are plotted as a continuous function of analysis time, defined as time since the index self-harm event for individuals with self-harm or baseline for those without self-harm event. Separate models were fitted by sex and adjusted for age. Shaded areas indicate 95% confidence intervals. The dashed horizontal line represents a hazard ratio of 1, indicating no difference in risk. Fig C. Incidence of self-harm by age and sex, restricted to medical scheme data. Incidence per 100,000 person-years in general population as a continuous function of age, stratified by sex. Individuals were censored at the time of their first documented self-harm event. Repeated self-harm events were not considered. Fig D. Cumulative incidence of self-harm by sex and age group, restricted to medical scheme data. Aalen–Johansen estimate with 95% confidence intervals for c [file pmed.1004765.s004.docx]

Incidence and Prognostic Factors of Self-Harm and Subsequent Unnatural Death in South Africa: A Cohort Study

Supplementary Texts, Tables and Figures

**Table of Contents**

[Text A. Methodological details for post-hoc analysis 3](#_Toc208559951)

[Text B. Detailed results from sensitivity analyses restricted to medical scheme data 4](#_Toc208559952)

[Text C. Detailed results from sensitivity analyses comparing individuals included and excluded from mortality analyses 5](#_Toc208559953)

[Figure A: Incidence of self-harm by age, sex and HIV status 6](#_Toc208559954)

[Figure B. Time-varying hazard ratios for unnatural death by sex, comparing individuals with and without prior non-fatal self-harm encounter 7](#_Toc208559955)

[Figure C: Incidence of self-harm by age, and sex, restricted to medical scheme data 8](#_Toc208559956)

[Figure D. Cumulative incidence of self-harm by sex and age group, restricted to medical scheme data 9](#_Toc208559957)

[Figure E. Predictors of self-harm, restricted to medical scheme data 10](#_Toc208559958)

[Figure F. Hazard ratios for unnatural death comparing individuals with and without a history of non-fatal self-harm by sex, restricted to medical scheme data 11](#_Toc208559959)

[Figure G. Hazard ratios for self-harm method as a predictor of unnatural death, by sex, restricted to medical scheme data 12](#_Toc208559960)

[Table A. Incidence rates of self-harm encounters per 100,000 person-years, stratified by sex, for different age groups and overall 13](#_Toc208559961)

[Table B. Cumulative incidence (%) and 95% confidence intervals of self-harm, stratified by sex, for different age groups and overall 14](#_Toc208559962)

[Table C: Healthcare utilization before self-harm events compared with matched controls prior to hypothetical matched event dates 15](#_Toc208559963)

[Table D. Cumulative incidence (%) and 95% confidence intervals of unnatural death, stratified by sex and self-harm method, and overall 16](#_Toc208559964)

[Table E. Characteristics of the study population by cohort 17](#_Toc208559965)

[Table F. Characteristics of individuals included and excluded from the mortality analysis 18](#_Toc208559966)

# Text A. Methodological details for post-hoc analysis

*Post-hoc analyses*

We evaluated the lethality of the self-harm method at the index self-harm event as a predictor of subsequent unnatural death, estimating age-adjusted and fully adjusted hazard ratios for individuals using highly lethal versus less lethal methods, compared to those with no prior non-fatal self-harm encounter. Next, we used Royston–Parmar flexible parametric survival models [1] (3 degrees of freedom (df) spline, stratified by sex and adjusted for age using a natural spline (3 df), to estimate age-adjusted, time-varying hazard ratios for non-fatal intentional self-harm, modelled as a time-dependent predictor.

**References**

1. Royston P, Parmar MKB. Flexible parametric proportional-hazards and proportional-odds models for censored survival data, with application to prognostic modelling and estimation of treatment effects. Stat Med. 2002;21: 2175–2197. doi:10.1002/sim.1203

# Text B. Detailed results from sensitivity analyses restricted to medical scheme data

Compared with the medical scheme cohort, the HIV cohort included a higher proportion of females (59.0% vs 51.8%) and fewer individuals aged under 25 years (11.1% vs 32.3%). The proportions diagnosed with mental disorders, and the proportion who died during follow-up, were higher in the HIV program cohort. However, these estimates are not directly comparable due to differing follow-up durations (median 9.0 years in the HIV cohort vs 3.1 years in the medical scheme cohort) (Table E).

In sensitivity analyses restricted to the medical insurance cohort, findings on the incidence of self-harm, its predictors, and non-fatal self-harm as a predictor of unnatural death were consistent with the primary analysis, although strength of associations changed somewhat. Specifically, the incidence of intentional self-harm was largely unchanged: among males, the incidence remained at 90 per 100,000 person-years (95% CI 86–94); among females, it decreased slightly from 183 to 180 per 100,000 person-years (95% CI 174–185). Age-specific incidence functions (Figure C) and cumulative incidence curves (Figure D) closely resembled those observed in the primary analysis. The association between a positive HIV status and self-harm incidence was modestly stronger (HR 1.32, 95% CI 1.22–1.44) (Figure E) compared with the estimate from the primary analysis (HR 1.23 CI 1.16–1.30). Associations between mental disorders and the incidence of self-harm remained stable in the sensitivity analysis, with only minor changes in the strength of associations (Figure E). Associations between prior non-fatal self-harm and subsequent unnatural death were slightly stronger (Figure F).

Findings were not robust, however, in sensitivity analyses of self-harm method as a predictor of unnatural death. Among females, associations with highly lethal methods were stronger in the medical scheme cohort (age-adjusted HR 40.50, 95% CI 10.10–162.00; Figure G) compared with the primary analysis (HR 29.2, 95% CI 7.29–117.0). Among males, use of highly lethal methods at the index self-harm encounter was no longer a statistically significant predictor of subsequent unnatural death (age-adjusted HR 5.05, 95% CI 0.71–35.90; Figure G), due to the exclusion of several male deaths involving highly lethal methods that occurred in the HIV program.

# Text C. Detailed results from sensitivity analyses comparing individuals included and excluded from mortality analyses

Excluded individuals were younger (mean age 25.3 vs 35.1 years; standardized difference 0.61), had a slightly higher HIV prevalence (16.2% vs 12.8%; standardized difference 0.10), and slightly lower prevalence of depression (9.0% vs 13.8%; standardized difference 0.15) and anxiety (11.6% vs 16.5%; standardized difference 0.14) compared with those included in the analysis (Table F). Self-harm incidence was higher among excluded individuals compared with those included in the mortality analysis, in models adjusted for age group, sex, HIV status, and mental disorders (HR 1.25, 95% CI 1.15–1.36).

# Figure A: Incidence of self-harm by age, sex and HIV status

Incidence per 100,000 person-years in general population as a continuous function of age, stratified by sex and HIV status. Individuals were censored at the time of their first documented self-harm event. Repeated self-harm events were not considered.


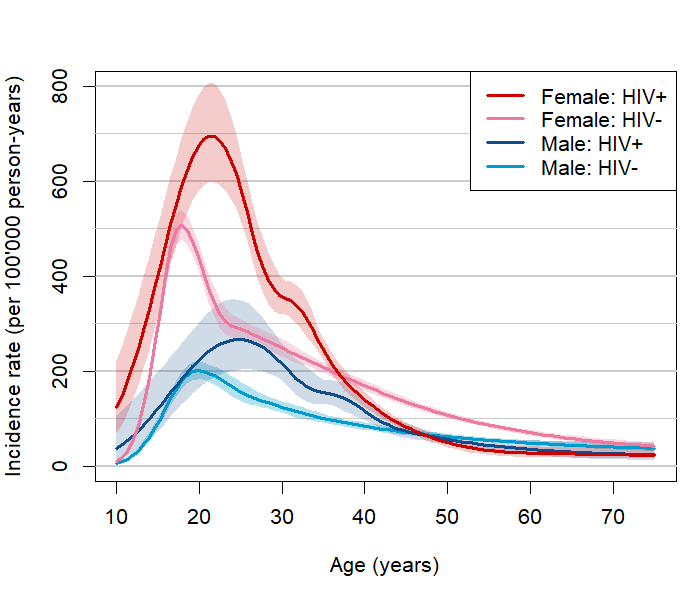


# Figure B. Time-varying hazard ratios for unnatural death by sex, comparing individuals with and without prior non-fatal self-harm encounter

Hazard ratios are plotted as a continuous function of analysis time, defined as time since the index self-harm event for individuals with self-harm or baseline for those without self-harm event. Separate models were fitted by sex and adjusted for age. Shaded areas indicate 95% confidence intervals. The dashed horizontal line represents a hazard ratio of 1, indicating no difference in risk.
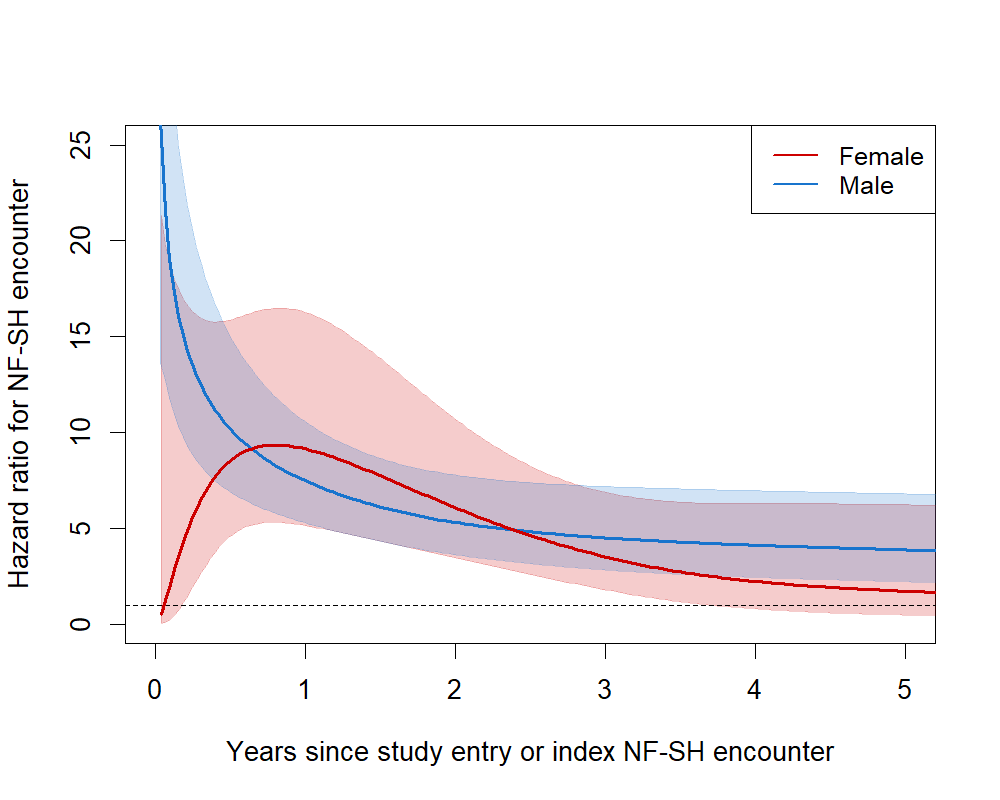


# Figure C: Incidence of self-harm by age, and sex, restricted to medical scheme data

Incidence per 100,000 person-years in general population as a continuous function of age, stratified by sex. Individuals were censored at the time of their first documented self-harm event. Repeated self-harm events were not considered.

**
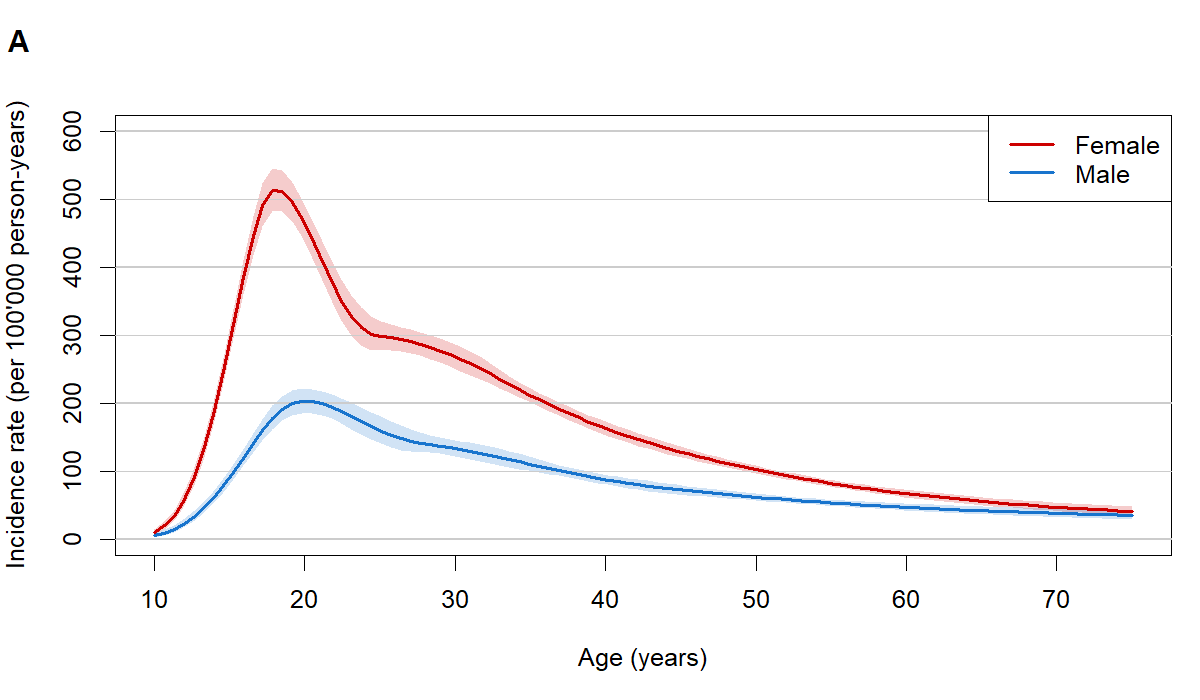
**

# Figure D. Cumulative incidence of self-harm by sex and age group, restricted to medical scheme data

Aalen-Johansen estimate with 95% confidence intervals for cause-specific cumulative over time since baseline or age-group change, stratified by sex and age group. Individuals were censored at the time of their first documented self-harm event. Repeated self-harm events were not considered.

**
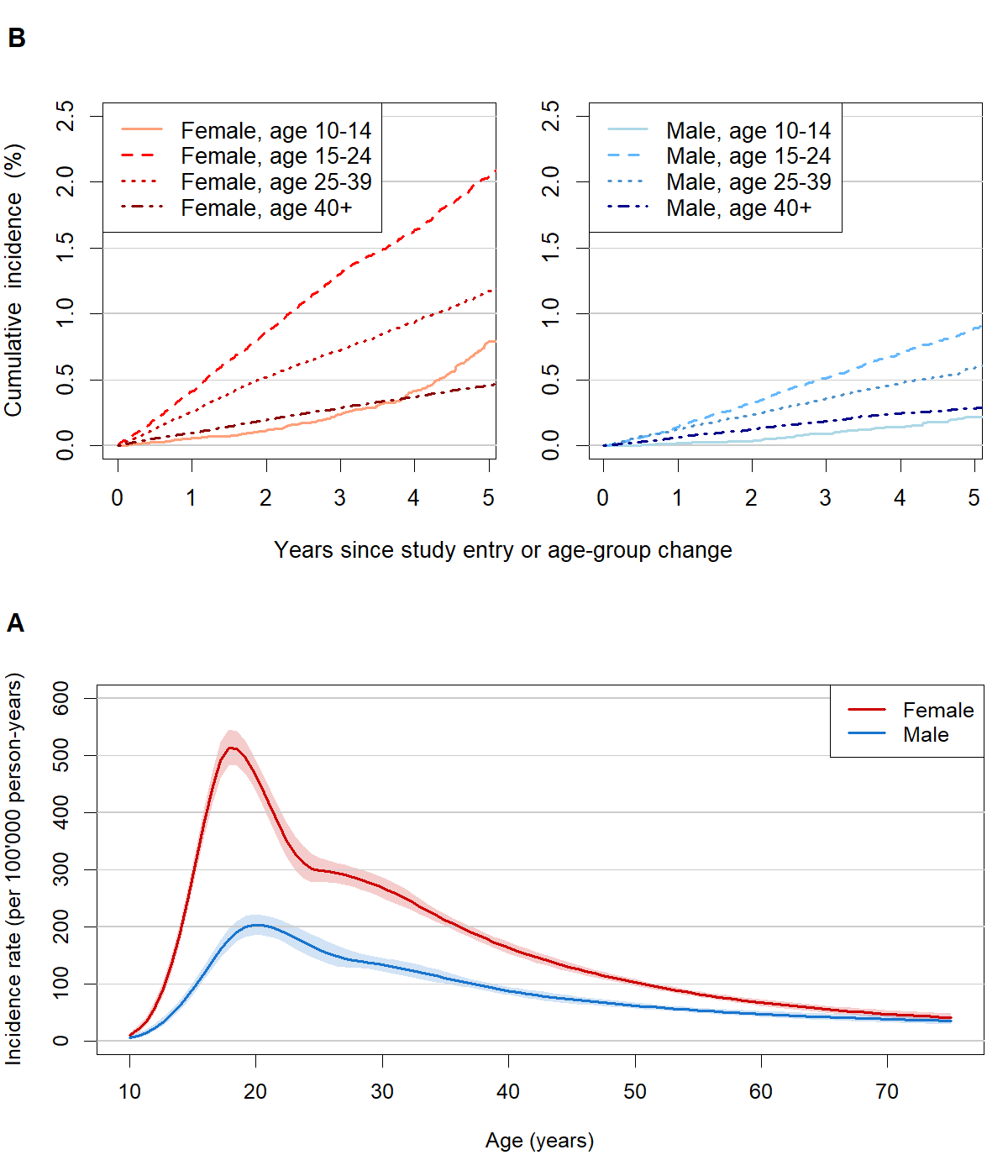
**

# Figure E. Predictors of self-harm, restricted to medical scheme data

The figure shows age- and sex-adjusted and fully adjusted hazard ratios for predictors of self-harm. Individuals were censored at the time of their first documented self-harm event. Repeated self-harm events were not considered. The error bars represent 95% confidence intervals. Fully adjusted models controlled for age group, sex, HIV and all mental disorders.


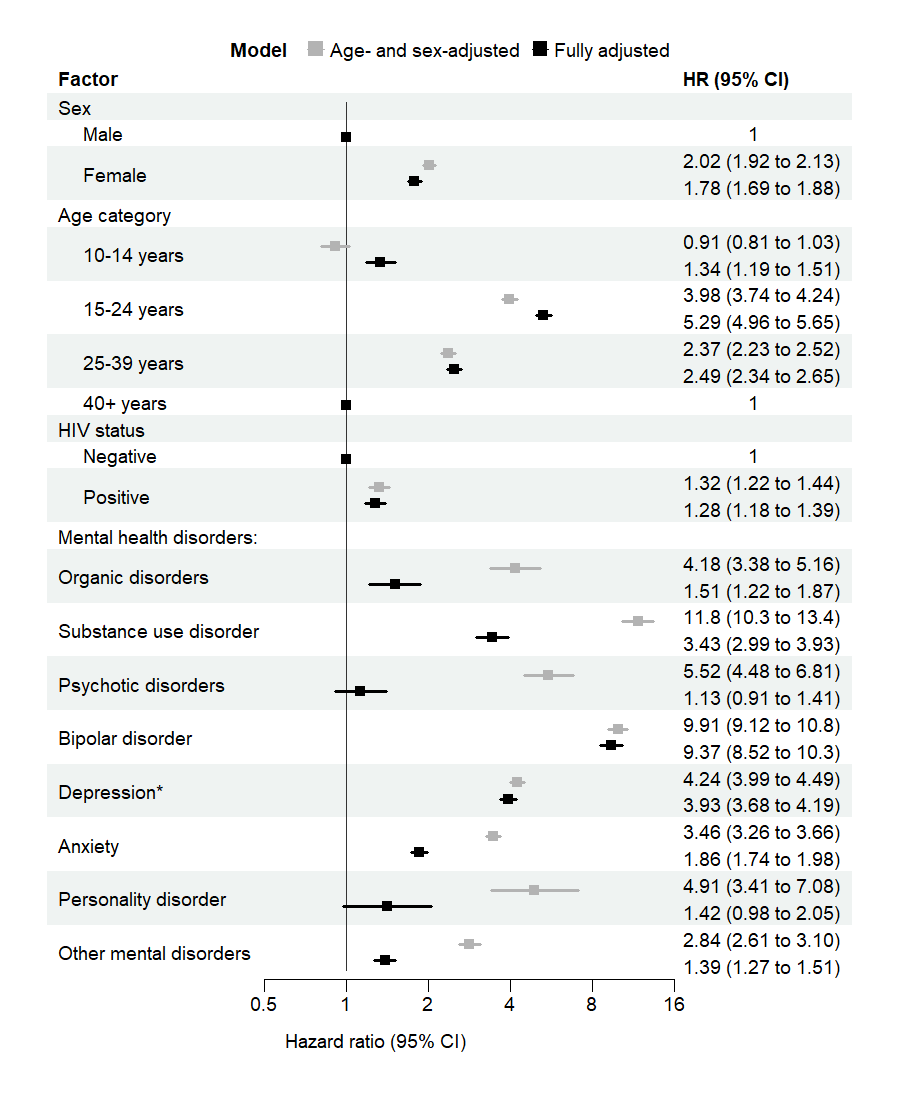


* For individuals diagnosed with both depression and bipolar disorder, only the bipolar disorder diagnosis was considered from the date of bipolar diagnosis onwards.

Abbreviations: CI, confidence intervals; HR, hazard ratio

# Figure F. Hazard ratios for unnatural death comparing individuals with and without a history of non-fatal self-harm by sex, restricted to medical scheme data

All models were fitted separately for males and females. Age-adjusted estimates control for age using it as the timeline in Cox model. Fully adjusted models additionally control for HIV status, mental health diagnoses. Error bars represent 95% confidence intervals.

**
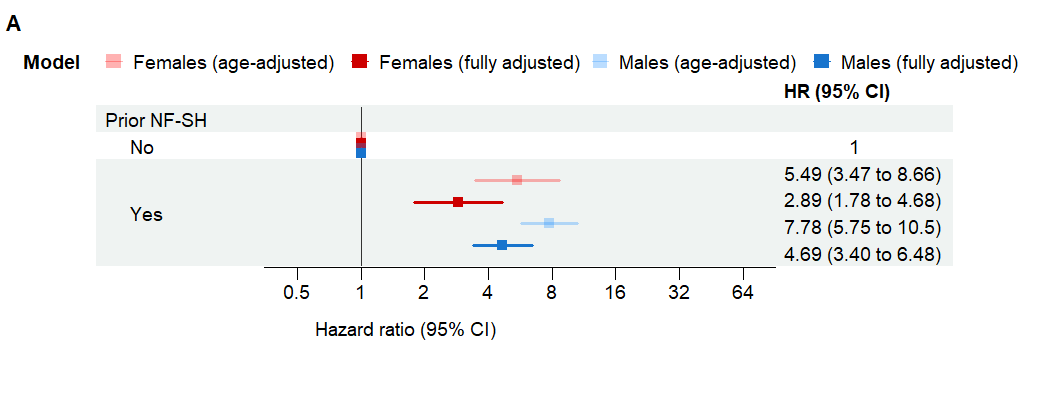
**

Abbreviations: NF-SH, non-fatal self-harm; CI, confidence intervals; HR, hazard ratio

# Figure G. Hazard ratios for self-harm method as a predictor of unnatural death, by sex, restricted to medical scheme data

All models were fitted separately for males and females. Hazard ratios compare individuals who used highly lethal or less lethal methods at their index self-harm encounter with individuals without a history of non-fatal self-harm (NF-SH). Age-adjusted estimates control for age by using it as the timeline in the Cox model. Fully adjusted models additionally control for HIV status and mental health diagnoses. Error bars represent 95% confidence intervals.

**
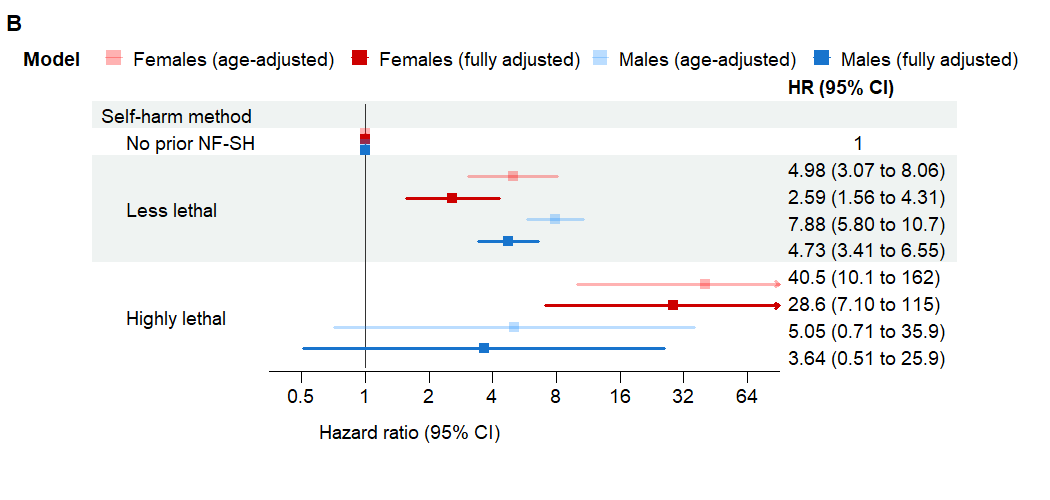
**

Abbreviations: NF-SH, non-fatal self-harm; CI, confidence intervals; HR, hazard ratio

# Table A. Incidence rates of self-harm encounters per 100,000 person-years, stratified by sex, for different age groups and overall

|  | **Female** | | |  | **Male** | | |
| --- | --- | --- | --- | --- | --- | --- | --- |
|  | Events | Person-years | Rate (95% CI) |  | Events | Person-years | Rate (95% CI) |
| Age, years |  |  |  |  |  |  |  |
| 10-14 | 261 | 248213.9 | 105.2 (92.4,118) |  | 83 | 253460.6 | 32.7 (25.7,39.7) |
| 15-24 | 1663 | 391616.4 | 424.7 (404.3,445.1) |  | 633 | 365352.9 | 173.3 (159.8,186.8) |
| 25-39 | 2089 | 856862.4 | 243.8 (233.3,254.3) |  | 869 | 708882.1 | 122.6 (114.4,130.8) |
| 40+ | 1195 | 1342932.9 | 89 (84,94) |  | 717 | 1223354 | 58.6 (54.3,62.9) |
| All ages | 5208 | 2839625.4 | 183.4 (178.4,188.4) |  | 2302 | 2551049.7 | 90.2 (86.5,93.9) |

Abbreviations: CI, confidence intervals. Individuals were censored at the time of their first documented self-harm event. Repeated self-harm events were not considered.

# Table B. Cumulative incidence (%) and 95% confidence intervals of self-harm, stratified by sex, for different age groups and overall

|  | **Time since baseline or age category change, years** | | | | |
| --- | --- | --- | --- | --- | --- |
|  | 1 | 2 | 3 | 4 | 5 |
| **Female** |  |  |  |  |  |
| Age group, years |  |  |  |  |  |
| 10-14 | 0.06 (0.04,0.08) | 0.11 (0.09,0.14) | 0.24 (0.20,0.28) | 0.41 (0.35,0.48) | 0.80 (0.69,0.92) |
| 15-24 | 0.41 (0.37,0.44) | 0.87 (0.81,0.92) | 1.32 (1.25,1.40) | 1.67 (1.58,1.76) | 2.09 (1.97,2.21) |
| 25-39 | 0.25 (0.23,0.27) | 0.52 (0.49,0.55) | 0.75 (0.71,0.79) | 0.97 (0.92,1.02) | 1.23 (1.17,1.29) |
| 40+ | 0.10 (0.09,0.11) | 0.19 (0.18,0.21) | 0.29 (0.26,0.31) | 0.37 (0.34,0.39) | 0.45 (0.42,0.48) |
| All ages | 0.18 (0.17,0.19) | 0.37 (0.36,0.39) | 0.55 (0.53,0.57) | 0.72 (0.70,0.75) | 0.92 (0.89,0.95) |
| **Male** |  |  |  |  |  |
| Age group, years |  |  |  |  |  |
| 10-14 | 0.02 (0.01,0.03) | 0.04 (0.02,0.05) | 0.09 (0.07,0.12) | 0.14 (0.11,0.18) | 0.21 (0.17,0.27) |
| 15-24 | 0.14 (0.12,0.16) | 0.33 (0.30,0.37) | 0.52 (0.47,0.57) | 0.70 (0.64,0.77) | 0.91 (0.82,0.99) |
| 25-39 | 0.12 (0.11,0.14) | 0.24 (0.22,0.26) | 0.37 (0.34,0.41) | 0.49 (0.45,0.53) | 0.61 (0.56,0.66) |
| 40+ | 0.06 (0.05,0.07) | 0.12 (0.11,0.13) | 0.18 (0.17,0.20) | 0.24 (0.22,0.26) | 0.29 (0.26,0.31) |
| All ages | 0.08 (0.08,0.09) | 0.17 (0.16,0.18) | 0.27 (0.25,0.28) | 0.36 (0.34,0.38) | 0.45 (0.43,0.47) |

Aalen-Johansen estimates of cause-specific cumulative incidence of self-harm in percent and 95% confidence intervals. Individuals were censored at the time of their first documented self-harm event. Repeated self-harm events were not considered. Deaths were considered competing events.

# Table C: Healthcare utilization before self-harm events compared with matched controls prior to hypothetical matched event dates

|  | **Self-harm** | **Control** | **Total** | **Std. diff.** |
| --- | --- | --- | --- | --- |
|  | N=7,510 (33.3%) | N=15,020 (66.7%) | N=22,530 (100.0%) |  |
| Matching characteristics |  |  |  |  |
| Age at baseline, years |  |  |  |  |
| 10-14 | 1,281 (17.1) | 2,562 (17.1) | 3,843 (17.1) | 0.00 |
| 15-19 | 957 (12.7) | 1,914 (12.7) | 2,871 (12.7) | 0.00 |
| 20-39 | 3,742 (49.8) | 7,484 (49.8) | 11,226 (49.8) | 0.00 |
| 40-59 | 1,297 (17.3) | 2,594 (17.3) | 3,891 (17.3) | 0.00 |
| 60+ | 233 (3.1) | 466 (3.1) | 699 (3.1) | 0.00 |
| Mean (SD) | 28.7 (13.8) | 29.6 (14.4) | 29.3 (14.3) |  |
| Sex |  |  |  |  |
| Male | 2,302 (30.7) | 4,604 (30.7) | 6,906 (30.7) | 0.00 |
| Female | 5,208 (69.3) | 10,416 (69.3) | 15,624 (69.3) | 0.00 |
| Baseline year |  |  |  |  |
| 2011 | 4,342 (57.8) | 8,684 (57.8) | 13,026 (57.8) | 0.00 |
| 2012-2016 | 2,265 (30.2) | 4,530 (30.2) | 6,795 (30.2) | 0.00 |
| 2017-2021 | 903 (12.0) | 1,806 (12.0) | 2,709 (12.0) | 0.00 |
| Time from baseline to event, years |  |  |  |  |
| <2 | 868 (11.6) | 1,736 (11.6) | 2,604 (11.6) | 0.00 |
| 2-4 | 1,503 (20.0) | 3,006 (20.0) | 4,509 (20.0) | 0.00 |
| 4-6 | 1,288 (17.2) | 2,576 (17.2) | 3,864 (17.2) | 0.00 |
| 6 | 1,371 (18.3) | 2,742 (18.3) | 4,113 (18.3) | 0.00 |
| >8 | 2,480 (33.0) | 4,960 (33.0) | 7,440 (33.0) | 0.00 |
| Mean (SD) | 3.1 (2.3) | 3.1 (2.3) | 3.1 (2.3) |  |
| Health care encounters within 7 days before event |  |  |  |  |
| Under follow-up ≥7 days before event: denominator | 7,483 (100.0) | 14,945 (100.0) | 22,428 (100.0) |  |
| Any health care encounter | 2,656 (35.5) | 2,549 (17.1) | 5,205 (23.2) | 0.43 |
| OPD | 2,590 (34.6) | 2,506 (16.8) | 5,096 (22.7) | 0.42 |
| Hospital | 189 (2.5) | 83 (0.6) | 272 (1.2) | 0.16 |
| Mental health encounter | 667 (8.9) | 118 (0.8) | 785 (3.5) | 0.39 |
| OPD | 633 (8.5) | 117 (0.8) | 750 (3.3) | 0.37 |
| Hospital | 51 (0.7) | 4 (0.0) | 55 (0.2) | 0.11 |
| Psychiatric medication claim | 606 (8.2) | 159 (1.1) | 765 (3.5) | 0.34 |
| Antidepressants | 376 (5.1) | 106 (0.7) | 482 (2.2) | 0.26 |
| Anxiolytics | 283 (3.8) | 56 (0.4) | 339 (1.5) | 0.24 |
| Antipsychotics | 148 (2.0) | 20 (0.1) | 168 (0.8) | 0.18 |
| Health care encounters within 30 days before event |  |  |  |  |
| Under follow-up ≥30 days before event: denominator | 7,379 (100.0) | 14,660 (100.0) | 22,039 (100.0) |  |
| Any health care encounter | 5,158 (69.9) | 7,123 (48.6) | 12,281 (55.7) | 0.44 |
| OPD | 5,118 (69.4) | 7,071 (48.2) | 12,189 (55.3) | 0.44 |
| Hospital | 593 (8.0) | 291 (2.0) | 884 (4.0) | 0.28 |
| Mental health encounter | 1,425 (19.3) | 398 (2.7) | 1,823 (8.3) | 0.55 |
| OPD | 1,367 (18.5) | 396 (2.7) | 1,763 (8.0) | 0.53 |
| Hospital | 216 (2.9) | 16 (0.1) | 232 (1.1) | 0.23 |
| Psychiatric medication claim | 1,591 (21.6) | 642 (4.4) | 2,233 (10.1) | 0.53 |
| Antidepressants | 1,150 (15.6) | 444 (3.0) | 1,594 (7.2) | 0.44 |
| Anxiolytics | 749 (10.2) | 226 (1.5) | 975 (4.4) | 0.37 |
| Antipsychotics | 465 (6.3) | 108 (0.7) | 573 (2.6) | 0.31 |
| Health care encounters within 90 days before event |  |  |  |  |
| Under follow-up ≥90 days before event: denominator | 7,081 (100.0) | 13,940 (100.0) | 21,021 (100.0) |  |
| Any health care encounter | 6,134 (86.6) | 9,946 (71.3) | 16,080 (76.5) | 0.38 |
| OPD | 6,106 (86.2) | 9,886 (70.9) | 15,992 (76.1) | 0.38 |
| Hospital | 1,141 (16.1) | 805 (5.8) | 1,946 (9.3) | 0.34 |
| Mental health encounter | 1,964 (27.7) | 720 (5.2) | 2,684 (12.8) | 0.64 |
| OPD | 1,901 (26.8) | 708 (5.1) | 2,609 (12.4) | 0.62 |
| Hospital | 432 (6.1) | 49 (0.4) | 481 (2.3) | 0.33 |
| Psychiatric medication claim | 2,143 (30.3) | 1,081 (7.8) | 3,224 (15.3) | 0.60 |
| Antidepressants | 1,571 (22.2) | 702 (5.0) | 2,273 (10.8) | 0.52 |
| Anxiolytics | 1,116 (15.8) | 437 (3.1) | 1,553 (7.4) | 0.44 |
| Antipsychotics | 649 (9.2) | 188 (1.3) | 837 (4.0) | 0.36 |

Abbreviations: SD, standard deviation; OPD, outpatient department

# Table D. Cumulative incidence (%) and 95% confidence intervals of unnatural death, stratified by sex and self-harm method, and overall

|  | **Time since baseline or self-harm encounter, years** | | | | |
| --- | --- | --- | --- | --- | --- |
|  | 1 | 2 | 3 | 4 | 5 |
| **Female** |  |  |  |  |  |
| No previous non-fatal intentional self-harm encounter | 0.03 (0.03,0.03) | 0.06 (0.05,0.06) | 0.09 (0.08,0.10) | 0.12 (0.11,0.13) | 0.15 (0.14,0.16) |
| Previous non-fatal intentional self-harm encounter | 0.20 (0.09,0.38) | 0.35 (0.20,0.60) | 0.59 (0.36,0.93) | 0.77 (0.48,1.19) | 0.77 (0.48,1.19) |
| Self-harm method |  |  |  |  |  |
| Less lethal | 0.18 (0.08,0.35) | 0.31 (0.16,0.54) | 0.54 (0.32,0.89) | 0.73 (0.44,1.15) | 0.73 (0.44,1.15) |
| Highly lethal | 1.96 (0.15,9.19) | 4.26 (0.76,13.00) | 4.26 (0.76,13.00) | 4.26 (0.76,13.00) | 4.26 (0.76,13.00) |
| **Male** |  |  |  |  |  |
| No previous non-fatal intentional self-harm encounter | 0.11 (0.10,0.12) | 0.21 (0.20,0.23) | 0.32 (0.30,0.33) | 0.41 (0.39,0.43) | 0.51 (0.49,0.54) |
| Previous non-fatal intentional self-harm encounter | 1.52 (1.03,2.16) | 1.90 (1.32,2.63) | 2.46 (1.74,3.38) | 2.74 (1.94,3.75) | 3.43 (2.38,4.76) |
| Self-harm method |  |  |  |  |  |
| Less lethal | 1.42 (0.94,2.07) | 1.82 (1.25,2.56) | 2.41 (1.68,3.35) | 2.70 (1.88,3.73) | 3.41 (2.33,4.79) |
| Highly lethal | 3.84 (1.01,9.89) | 3.84 (1.01,9.89) | 3.84 (1.01,9.89) | 3.84 (1.01,9.89) | 3.84 (1.01,9.89) |
| **Both sexes** |  |  |  |  |  |
| No previous non-fatal intentional self-harm encounter | 0.07 (0.06,0.07) | 0.13 (0.13,0.14) | 0.20 (0.19,0.21) | 0.26 (0.25,0.27) | 0.32 (0.31,0.34) |
| Previous non-fatal intentional self-harm encounter | 0.60 (0.43,0.82) | 0.82 (0.61,1.09) | 1.15 (0.87,1.51) | 1.37 (1.04,1.77) | 1.57 (1.18,2.05) |
| Self-harm method |  |  |  |  |  |
| Less lethal | 0.55 (0.38,0.76) | 0.75 (0.55,1.02) | 1.09 (0.81,1.44) | 1.31 (0.98,1.72) | 1.52 (1.13,2.01) |
| Highly lethal | 3.10 (1.01,7.22) | 4.05 (1.50,8.65) | 4.05 (1.50,8.65) | 4.05 (1.50,8.65) | 4.05 (1.50,8.65) |

Data are Aalen-Johansen estimates of cause-specific cumulative incidence of unnatural death. Deaths from natural and unknown causes were considered competing events.

# Table E. Characteristics of the study population by cohort

|  | **Medical scheme** | **HIV program** | **Total** |
| --- | --- | --- | --- |
|  | N=1,267,457 (93.5%) | N=88,662  (6.5%) | N=1,356,119  (100.0%) |
| **Characteristics at baseline** |  |  |  |
| Sex |  |  |  |
| Female | 656,742 (51.8%) | 52,331 (59.0%) | 709,073 (52.3%) |
| Male | 610,715 (48.2%) | 36,331 (41.0%) | 647,046 (47.7%) |
| Age, years |  |  |  |
| 10-14 | 209,015 (16.5%) | 2,992 (3.4%) | 212,007 (15.6%) |
| 15-24 | 199,828 (15.8%) | 6,817 (7.7%) | 206,645 (15.2%) |
| 25-39 | 393,786 (31.1%) | 48,358 (54.5%) | 442,144 (32.6%) |
| 40+ | 464,828 (36.7%) | 30,495 (34.4%) | 495,323 (36.5%) |
| Median (IQR) | 33 (20, 47) | 35 (29, 42) | 33.0 (21, 46) |
| **Characteristics at the end of follow-up*** |  |  |  |
| HIV status |  |  |  |
| HIV-negative | 1,178,926 (93.0%) | 0 (0.0%) | 1,178,926 (86.9%) |
| HIV-positive | 88,531 (7.0%) | 88,662 (100.0%) | 177,193 (13.1%) |
| Any mental health disorders | 306,446 (24.2%) | 41,711 (47.0%) | 348,157 (25.7%) |
| Organic mental disorder | 9,757 (0.8%) | 1,078 (1.2%) | 10,835 (0.8%) |
| Substance use disorder | 7,799 (0.6%) | 1,404 (1.6%) | 9,203 (0.7%) |
| Psychotic disorder | 4,696 (0.4%) | 786 (0.9%) | 5,482 (0.4%) |
| Bipolar disorder | 19,753 (1.6%) | 2,075 (2.3%) | 21,828 (1.6%) |
| Depression | 159,024 (12.5%) | 23,687 (26.7%) | 182,711 (13.5%) |
| Anxiety disorder | 189,069 (14.9%) | 29,512 (33.3%) | 218,581 (16.1%) |
| Personality disorder | 1,518 (0.1%) | 206 (0.2%) | 1,724 (0.1%) |
| Other mental health disorders | 59,886 (4.7%) | 7,851 (8.9%) | 67,737 (5.0%) |
| Self-harm encounters |  |  |  |
| Event of undetermined intent | 11,660 (0.9%) | 1,372 (1.5%) | 13,032 (1.0%) |
| Intentional self-harm | 6,540 (0.5%) | 970 (1.1%) | 7,510 (0.6%) |
| Follow-up time, years |  |  |  |
| Median (IRQ) | 3.1 (1.2,6.2) | 9.0 (4.7, 9.0) | 3.3 (1.2,6.8) |
| Linked to National Population Register | 1,181,450 (93.2%) | 75,238 (84.9%) | 1,256,688 (92.7%) |
| Died during follow-up** | 35,457 (3.0%) | 4,072 (5.4%) | 39,529 (3.1%) |
| Natural cause** | 30,786 (2.6%) | 3,484 (4.6%) | 34,270 (2.7%) |
| Unnatural cause** | 3,196 (0.3%) | 486 (0.6%) | 3,682 (0.3%) |
| Unknown cause** | 1,475 (0.1%) | 102 (0.1%) | 1,577 (0.1%) |

Data are n (%) unless otherwise stated. Abbreviations: IQR=interquartile range.
* Characteristics at end of follow-up are not directly comparable between groups due to difference in follow-up durations.

** Among those linked to National Population Register.

# Table F. Characteristics of individuals included and excluded from the mortality analysis

|  | **Included** | **Excluded** | **Total** | **Std. diff** |
| --- | --- | --- | --- | --- |
|  | N=1,256,623 (92.7%) | N=99,496 (7.3%) | N=1,356,119 (100.0%) |  |
| **Characteristics at baseline** |  |  |  |  |
| Sex |  |  |  |  |
| Male | 604,788 (48.1%) | 42,258 (42.5%) | 647,046 (47.7%) | 0.11 |
| Female | 651,835 (51.9%) | 57,238 (57.5%) | 709,073 (52.3%) | 0.11 |
| Age, years |  |  |  |  |
| 10-14 | 188,772 (15.0%) | 23,235 (23.4%) | 212,007 (15.6%) | 0.21 |
| 15-24 | 164,021 (13.1%) | 42,624 (42.8%) | 206,645 (15.2%) | 0.70 |
| 25-39 | 429,067 (34.1%) | 13,077 (13.1%) | 442,144 (32.6%) | 0.51 |
| 40+ | 474,763 (37.8%) | 20,560 (20.7%) | 495,323 (36.5%) | 0.38 |
| Mean (SD) | 35.1 (17.1) | 25.3 (14.7) | 34.4 (17.1) | 0.61 |
| **Characteristics at the end of follow-up*** |  |  |  |  |
| HIV status |  |  |  |  |
| HIV-negative | 1,095,591 (87.2%) | 83,335 (83.8%) | 1,178,926 (86.9%) | 0.10 |
| HIV-positive | 161,032 (12.8%) | 16,161 (16.2%) | 177,193 (13.1%) | 0.10 |
| Any mental disorder | 329,351 (26.2%) | 18,806 (18.9%) | 348,157 (25.7%) | 0.18 |
| Organic mental disorder | 10,449 (0.8%) | 386 (0.4%) | 10,835 (0.8%) | 0.06 |
| Substance use disorder | 8,606 (0.7%) | 597 (0.6%) | 9,203 (0.7%) | 0.01 |
| Psychotic disorder | 5,088 (0.4%) | 394 (0.4%) | 5,482 (0.4%) | 0.00 |
| Bipolar disorder | 20,786 (1.7%) | 1,042 (1.0%) | 21,828 (1.6%) | 0.05 |
| Depression | 173,765 (13.8%) | 8,946 (9.0%) | 182,711 (13.5%) | 0.15 |
| Anxiety disorder | 207,032 (16.5%) | 11,549 (11.6%) | 218,581 (16.1%) | 0.14 |
| Personality disorders | 1,609 (0.1%) | 115 (0.1%) | 1,724 (0.1%) | 0.00 |
| Other mental health disorders | 64,054 (5.1%) | 3,683 (3.7%) | 67,737 (5.0%) | 0.07 |
| Follow-up time, years |  |  |  |  |
| Mean (SD) | 4.0 (3.1) | 3.9 (3.0) | 4.0 (3.1) | 0.03 |

Data are n (%) unless otherwise stated. Abbreviations: SD=standard deviation.
