# Supplementary material for: Incidence and prognostic factors of self-harm and subsequent unnatural death in South Africa: A cohort study
Source: PLoS Med. 2025 Sep 26;22(9):e1004765. doi: 10.1371/journal.pmed.1004765 (PMC12503312; doi:10.1371/journal.pmed.1004765)
Supplement: Attachment — Submitted filename: PLOS Medicine May 2025.docx [file pmed.1004765.s005.docx]

Thank you for the opportunity to review this manuscript on a topic of clear importance. The methods are quite sound and the findings are striking and far-reaching, with potential for major impact in the field. I appreciated the extremely sounds methods and statistical approach and concise presentation of a variety of findings, but believe the manuscript would benefit from some clarifications and added detail in a few areas, outlined below.

1. Abstract: The focus on unnatural death (rather than death by suicide) is logical and the ICD codes used to define unnatural death are appropriate. However, a definition of what constitutes unnatural death would strengthen the abstract.
2. I appreciated the acknowledgement of some of the key limitations in the abstract.
3. Introduction: In paragraphs two and three, “Non-fatal suicidal behavior” seems to be used interchangeably with “suicide attempts”, which is not the case. Please address.
4. HIV status is considered in the methods and analytic plan but is completely absent from the abstract and introduction. In the prior literature, there is a link between living with HIV and suicide risk. This should be acknowledged and addressed.
5. Add considerations of how the sample of people who had health insurance coverage might differ from the general population.
6. Considerations of social determinants of health (e.g., food insecurity, housing insecurity, race/ethnicity, socioeconomic status) as predictors of unnatural death in this sample are completely absent. Why weren’t these considered and might it be beneficial to explore them in future studies?
7. It appears the second sub-sample were people living with HIV. Given the prior literature highlighting HIV status as a risk factor for suicide, the implications of using this sub-sample should be addressed more thoroughly. Tables S5 and S6 show higher risk among people with HIV but this is not addressed in the Abstract or Results – please add.
8. Did the prevalence of HIV in the final sample reflect the prevalence in the general population? If people living with HIV were over-sampled, how might this have impacted the generalizability of findings?
9. Similarly, comparison of baseline prevalence of common mental disorders in your sample with the prevalence in the general population will give some indication of generalizability.
