# Supplementary material for: Incidence and prognostic factors of self-harm and subsequent unnatural death in South Africa: A cohort study
Source: PLoS Med. 2025 Sep 26;22(9):e1004765. doi: 10.1371/journal.pmed.1004765 (PMC12503312; doi:10.1371/journal.pmed.1004765)
Supplement: Attachment — Submitted filename: Responses_to_reviewers_v1.1.docx [file pmed.1004765.s006.docx]

Response to Editors’ and Reviewers’ Comments

We thank the editors and reviewers for their thoughtful and constructive feedback, which has helped us to substantially improve the quality and clarity of our manuscript.

We have carefully revised the manuscript, with particular attention to: (1) improving the framing of the study within the broader self-harm literature; (2) clearly articulating the study’s prognostic aim; (3) refining the interpretation of estimates; (4) clarifying the clinical and policy implications of our findings; and (5) more rigorously assessing potential biases and generalizability in several sensitivity analyses.

Following the editorial guidance, we have retained a single, integrated manuscript rather than splitting it into several papers. We believe the combined presentation of our analyses offers a coherent and policy-relevant contribution to self-harm and suicide prevention in South Africa. The self-harm analysis provides local incidence data and identifies predictors for self-harm to guide early identification efforts such as targeted screening strategies. The mortality analysis informs the clinical management of individuals presenting with self-harm, a clearly defined high-risk group in need for suicide-specific interventions such as safety planning with structured follow-up care. Both approaches are aligned with WHO’s *LIVE LIFE* framework and are recognized as key effective suicide prevention strategies [1].

We respond to each comment in detail below. Our responses are shown in blue; quotes from the manuscript are indented. Revisions to the manuscript and supplementary materials are highlighted accordingly. Changes related to the prognostic nature of the study are marked in blue. Other substantive changes are marked in yellow. Minor language edits (e.g. for conciseness) are not highlighted.

We thank you again for your careful review and look forward to your feedback on the revised submission.

Sincerely,

Andreas Haas (on behalf of all co-authors)

-----------------------------------------------------------
Comments from the academic editor:

1.      Intentional self-harm is not necessarily suicidal so should not be considered synonymous with a “suicide attempt” (e.g. in the abstract) or suicidal behaviour (background). The authors need to engage with the intentional self-harm literature in the background and explain how it relates to suicidal intentions and attempts in this context. For example, cutting in high-income countries is described as ‘intentional self-harm’ but is conceptualised as a maladaptive coping strategy for emotional regulation and rarely suicidal. In the findings from this paper, cutting seems to be uncommon as a means of intentional self-harm so the authors can make the argument that is more closely linked to suicidal intention.

Thank you for this important comment. We have rewritten our introduction to frame the paper within the literature on self-harm. We begin by defining the spectrum of self-harm behaviors, including non-suicidal self-injury, suicide attempts, and suicide. We then review the literature on the prevalence of these behaviors in South Africa. Next, we summarize findings on self-harm presentations to emergency settings in South Africa to highlight that, in this context and in our study, non-suicidal self-injury plays a minor role. Most self-harm cases presenting to hospital settings in South Africa involve self-poisoning without documented suicidal intent. In the following paragraph, we discuss evidence that all individuals with a history of self-harm, not only those with suicide attempts, are at increased risk of repeated self-harm and unnatural death.

Below are the revised first four paragraphs of the introduction, reflecting these changes and incorporating the suggested reframing within the intentional self-harm literature.

“Intentional self-harm (hereafter referred to as self-harm) includes a spectrum of behaviors in which individuals deliberately harm themselves. The literature distinguishes three types of self-harm behaviors: non-suicidal self-injury, defined as deliberate, directly injurious behavior (e.g. cutting, or burning) without any intent to die; suicide attempts, defined as non-fatal potentially self-injurious behaviors with at least some intent to die; and suicide, defined as fatal self-directed injurious behavior with intent to die [1,2].

Self-harm contributes substantially to the global disease burden and places considerable strain on health systems, especially in low- and middle-income countries like South Africa, where self-harm rates are high [3,4] and resources for prevention and care are scarce [5,6]. The crude suicide rate in South Africa in 2021 was estimated at 35.4 per 100,000 population among males, 9.9 among females, placing the country among those with the highest rates globally [7]. Lifetime prevalence of suicide attempts in South Africa is 3.8% among females and 1.8% among males [8]. Non-suicidal self-injury, which predominantly affects adolescents and young adults [1,2], is also highly prevalent with lifetime estimates ranging from 33% to 56% [9,10].

Self-harm cases presenting to emergency departments in South Africa are predominantly episodes of self-poisoning without evidence of suicidal intent or suicide attempts. At hospitals in Cape Town and KwaZulu-Natal, poisoning, mainly with medication, accounted for 72-80% of presentations, while highly lethal methods such as hanging or laceration were less common (6–14%). Across all methods, most patients (66–78%) reported no intent to die [11,12]. While non-suicidal self-injury is common among adolescents in South Africa, it is infrequently seen in hospital or emergency settings [11,12].

Self-harm behaviors are often recurrent and may escalate in severity [2,13]. Individuals with a history of self-harm are at increased risk of unnatural death including suicide and accidental death [14–17]. Non-suicidal self-injury has consistently been shown to predict suicidal ideation and suicide attempts [18]. Intentional self-poisoning, regardless of intent, is a strong predictor subsequent suicide [14]. The risk of recurrent suicidal behavior after a suicide attempt is well documented, with 16% reattempting and 3% dying by suicide within one year [13,15]. Beyond suicide, individuals with a self-harm history also face increased risks of accidental, alcohol-related, and drug-related mortality [16,17].”

2.      How complete is the coverage of the national vital registration database?

South Africa is known for its well-functioning and reliable Civil Registration and Vital Statistics System (CRVS) with an estimated 93% of adult deaths registered [2]. We obtained mortality data from the National Population Register (NPR), maintained by the South African Medical Research Council (SAMRC). The completeness of the NPR is regularly assessed against the CRVS. During the study period, the registration of deaths among adults aged 25 years and older in the NPR was complete or exceeding 100% when compared to Stats SA’s CRVS. For other age groups, completeness ranged between 90% and 95%, except for infants under one year of age (not included in our study), where it was substantially lower [3].

To clarify the completeness of NPR data, we have added the following sentence to the Methods section (Data Sources):

“*Death registration is estimated to be 93% complete among adults and slightly lower among children [26,27].”*

3.      It is necessary to present characteristics of the two cohorts separately. The inclusion of a cohort based on HIV status will affect the findings – recommend that the stratified findings are presented in a supplementary file.

We appreciate the reviewers’ and editors’ suggestion to examine how the inclusion of the HIV program may have influenced the results and have thoroughly investigated this issue.

As suggested, we have added a descriptive table showing demographic and clinical characteristics stratified by cohort (medical scheme vs HIV program) (Table E in Appendix S1). As expected, females are overrepresented in the HIV program (59% vs 52% in the medical scheme), reflecting a higher HIV prevalence and treatment coverage among females. Individuals in the HIV program were also slightly older, including fewer children, adolescents, and young adults, consistent with lower HIV prevalence in these age groups.

The remaining characteristics in Table E reflect the prevalence of diagnosed health conditions by the end of follow-up. As expected, the prevalence of mental disorders and mortality was higher in the HIV cohort than in the general medical scheme population. However, these comparisons are limited by substantial differences in follow-up duration (median: 9 years in the HIV program vs 3 years in the medical scheme). These crude prevalence estimates do not account for person-time under follow-up and are therefore not directly comparable across groups with varying follow-up time. We have noted this limitation in a footnote to the table and in Text B, Appendix S1.

Beyond these descriptive comparisons, we conducted sensitivity analyses to assess the impact of oversampling people with HIV on study findings. Presenting all analyses stratified by cohort was not feasible, as many models could not be estimated in the HIV cohort alone due to a low number of events, and no variability in HIV status. Therefore, we instead repeated all primary analyses using only data from the medical scheme, excluding the HIV program.

We present sensitivity analyses restricted to medical scheme data, excluding HIV program in the manuscript:

Methods (Sensitivity analyses):

*“In post-hoc sensitivity analyses, we repeated all primary analyses in the medical scheme cohort only, to assess the impact of oversampling individuals with HIV due to the inclusion of HIV program data.”*

Results (Sensitivity analyses):

*“Compared with the medical scheme cohort, the HIV program cohort included a higher proportion of females and fewer individuals younger than 25 years (Table E in Appendix S1). Main findings remained consistent in sensitivity analyses restricted to the medical scheme cohort, although the strength of associations varied somewhat (Text B, Figures D-G in Appendix S1). One notable divergence was observed in the analysis of self-harm method as a predictor of unnatural death, where findings were not robust in sensitivity analyses: associations for highly lethal methods were stronger in females but attenuated and not statistically significant in males (Text B, Figure H in Appendix S1).”*

We provide a detailed account of these results in Text B, Table E, and Figures D-H:

*“***Text B. Detailed results from sensitivity analyses restricted to medical scheme data**

Compared with the medical scheme cohort, the HIV cohort included a higher proportion of females (59.0% vs 51.8%) and fewer individuals aged under 25 years (11.1% vs 32.3%). The proportions diagnosed with mental disorders, and the proportion who died during follow-up, were higher in the HIV program cohort. However, these estimates are not directly comparable due to differing follow-up durations (median 9.0 years in the HIV cohort vs 3.1 years in the medical scheme cohort) (Table E).

In sensitivity analyses restricted to the medical insurance cohort, findings on the incidence of self-harm, its predictors, and non-fatal self-harm as a predictor of unnatural death were consistent with the primary analysis, although strength of associations changed somewhat. Specifically, the incidence of intentional self-harm was largely unchanged: among males, the incidence remained at 90 per 100,000 person-years (95% CI 86–94); among females, it decreased slightly from 183 to 180 per 100,000 person-years (95% CI 174–185). Age-specific incidence functions (Figure D) and cumulative incidence curves (Figure E) closely resembled those observed in the primary analysis. The association between a positive HIV status and self-harm incidence was modestly stronger (HR 1.32, 95% CI 1.22–1.44) (Figure F) compared with the estimate from the primary analysis (HR 1.23 CI 1.16–1.30). Associations between mental disorders and the incidence of self-harm remained stable in the sensitivity analysis, with only minor changes in the strength of associations (Figure F). Associations between prior non-fatal self-harm and subsequent unnatural death were slightly stronger (Figure G).

Findings were not robust, however, in sensitivity analyses of self-harm method as a predictor of unnatural death. Among females, associations with highly lethal methods were stronger in the medical scheme cohort (age-adjusted HR 40.50, 95% CI 10.10–162.00; Figure H) compared with the primary analysis (HR 29.2, 95% CI 7.29–117.0). Among males, use of highly lethal methods at the index self-harm encounter was no longer a statistically significant predictor of subsequent unnatural death (age-adjusted HR 5.05, 95% CI 0.71–35.90; Figure H), due to the exclusion of several male deaths involving highly lethal methods that occurred in the HIV program.”

We have added the following text to the 7th paragraph of the Discussion to reflect on the results of the sensitivity analyses and their implications for interpreting our findings.

“Fourth, we combined data from the medical scheme and HIV program to increase sample size, resulting in oversampling of individuals with HIV, which may have introduced bias. Sensitivity analyses restricted to medical scheme data showed no minimal impact on self-harm incidence, its predictors, or its association with subsequent unnatural death, suggesting that oversampling did not materially affect the findings. However, in males, the association between highly lethal methods and unnatural death was not robust in sensitivity analysis, likely due to small numbers in the highly lethal exposure group. We therefore consider this finding exploratory and recommend replication. Nonetheless, results from the primary analysis are clinically plausible and consistent with a Swedish study [31], supporting their credibility.”

4.      Please change ‘completed suicide’ to ‘death by suicide’ in line with recommended language to reduce stigma.

Thank you for highlighting this important point. We are committed to using non-stigmatizing language and acknowledge that we missed this instance. We have made the change and now use the term “suicide,” without qualification in line with recommended terminology.

-----------------------------------------------------------
Comments from the reviewers:

**Reviewer #1** (statistics):
Thanks for the opportunity to read your manuscript. My role is statistical reviewer, so I have focused on the design, data, and analysis that are presented. I have put general comments first, followed by questions relevant to a specific section of the manuscript (with a page/paragraph reference).

This manuscript presents the results of a cohort study investigating incidence and risk factors of self-harm and unnatural deaths in South Africa. Data come from routinely collected sources, a private HIV management program, medical insurance scheme, and a national vital registration database. People aged 10 or over covered the insurance scheme between 2011 and 2020 were included. The main outcomes were the first coded healthcare record for intentional self-harm, and 'unnatural deaths'. A range of covariates including demographics and health status were available. For the mortality data, the primary exposure of interest was a healthcare record for non-fatal self-harm. Healthcare utilisation was compared between exposed and non-exposed, with matching on age and length of follow-up. Overall rates by age and sex were estimated from Poisson regression, and flexible parametric survival models were used for incidence of self-harm (with death as competing risk), including age as covariate and stratified by sex and HIV status. The approach to the analysis is sound (the FSPM is a good choice). I found this to be an interesting and well-written manuscript with well-presented results. I had some requests for clarification about the analysis and data sources, my primary concern was about the interpretation of the multivariable models.

We thank Reviewer #1 for their thoughtful statistical review and constructive suggestions. We have addressed all comments in detail and revised the manuscript accordingly. Revisions include a more detailed description of data sources, clarification of the study’s prognostic nature, consistent emphasis on the non-causal interpretation of estimates, and a clearer articulation of the clinical relevance and intended application of the identified predictors.

1.      It would help in understanding the coverage and limitations if some additional details about the datasets and linkage were provided. In particular, the eligibility and coverage of the medical insurance scheme, HIV disease management program, and vital registration scheme (particularly what information is available from this database). What method of data linkage (deterministic, SLK, probabilistic) was used and on what variables? Why were two databases (insurance + HIV management) used?

We have restructured and expanded the Methods section to include the requested additional details.

**Eligibility and coverage** of the medical scheme and HIV program are now described in the Methods section under “Setting.”

“The medical scheme is open to all South African residents but requires paid membership. Most residents do not have private insurance and rely on public sector services [28]. The HIV program includes individuals with HIV whose medical schemes have contracted the program to manage their HIV care. Medical scheme beneficiaries generally have higher socio-economic status and may face fewer psychosocial stressors of self-harm than individuals without insurance [29]. In addition to medical scheme benefits, all beneficiaries are entitled to Prescribed Minimum Benefits (PMBs), which include essential services. For self-harm, PMBs cover up to three days of inpatient care or six outpatient visits per episode, including treatment of injuries, complications, and underlying conditions [30].”

We clarified the **rationale for combining the two datasets** in the Methods under Data sources:

*“We combined data from the medical scheme and HIV program to increase sample size, given the rarity of unnatural death after self-harm.”*

**Vital registration data**: Mortality data were obtained from the National Population Register (NPR), which is maintained by the South African Medical Research Council (SAMRC). Linkage to mortality data was deterministic and based on the national identification number. Only individuals with a valid national ID were included in the mortality analyses. From the NPR, we obtained information on whether a death was recorded, the date of death, and cause of death, categorized as natural, unnatural, or unknown.

We clarified the available variables from the medical schemes, the NPR, and the linkage procedure in the Methods section under “Data sources”.

“We combined data from the medical scheme and HIV program to increase sample size, given the rarity of unnatural death after self-harm. The dataset included sociodemographic characteristics, reimbursement claims for inpatient and outpatient care with International Classification of Diseases (ICD-10) diagnoses, pharmacy claims, and laboratory results. These data were deterministically linked to vital registration records from the National Population Register using national identifiers […]. Vital registration data included date of death, and cause of death (natural, unnatural, or unknown).

2.      A limitation of some of the results (e.g. fig 2) with a multivariable model is that the effect estimates have different interpretations depending on the causal model that is assumed ('table 2 fallacy')

<https://academic.oup.com/aje/article-abstract/177/4/292/147738?redirectedFrom=fulltext>

Essentially the estimates will be a mix of 'direct-effects' and 'total-effects' which have different interpretations. I would consider either a) proposing a causal model so that the estimates can be interpreted correctly, b) a + concentrating on previous self-harm exposure (in mortality analysis), c) or looking at the data more descriptively.

Thank you for this comment, which helped us clarify that our objective was prognostic rather than etiologic. Our aim was to identify individuals at increased risk of future self-harm and unnatural death to inform suicide prevention efforts in South Africa, specifically, to support early detection of at-risk individuals in the general population and to guide safe and appropriate care for those presenting with self-harm. We did not seek to identify causal risk factors, i.e. variables that, when manipulated, alter the outcome [4,5]. Instead, we focused on prognostic factors (also referred to as predictive factors or risk markers), defined as characteristics measured before the outcome that indicate future risk, irrespective of causality [4,5]. We have revised the manuscript to clarify the prognostic aim, avoid causal language, and consistently refer to these variables as “predictors.” Relevant changes have been highlighted in light blue.

In the final paragraph of the Introduction, we now clearly state the prognostic nature of our study and clarify that estimates of predictors are intended to identify groups at increased risk and have no causal meaning.

“We aimed to estimate self-harm incidence, identify its predictors, and to evaluate non-fatal self-harm as a predictor of subsequent unnatural death. Our analyses were prognostic in nature and not designed to identify causal (etiologic) risk factors. Instead, we aimed to identify individual-level characteristics that serve as risk markers, indicating increased future self-harm or mortality risk, without necessarily implying causality [24,25].”

In the Methods section, we now refer to exposure variables as “predictors,” defined in the first sentence of the section as:

“We defined predictors as individual-level characteristics measured before the outcome that indicate increased future self-harm or mortality risk, without necessarily implying causality [24,25].”

In the first paragraph of the Discussion, we reiterate that our analyses were prognostic, not etiologic, and that the reported predictors should be interpreted as risk markers—useful for identifying individuals at elevated risk, not as causal risk factors.

“Key predictors of self-harm included young age, female sex, and mental health diagnoses. Non-fatal self-harm was a strong predictor of subsequent unnatural death in both sexes. As the analyses were prognostic in nature, the identified predictors should not be interpreted as causal but as risk markers to identify individuals at elevated risk.”

In the final paragraph of the Discussion, we emphasize their utility of the identified predictors in supporting early identification of individuals at risk of self-harm or in need for suicide-specific brief interventions.

“We identify strong predictors of self-harm that can guide targeted screening strategies, which could be implemented in general medical outpatient settings frequently visited in the weeks preceding self-harm—offering a critical opportunity for early detection and proactive management. Our finding that non-fatal self-harm is a strong risk marker for subsequent unnatural death highlights the need for indicated suicide prevention strategies to prevent recurrent self-harm and suicide in clearly defined high-risk populations.”

3.      Is it possible that some participants might be recurrent cases, with the data recording intentional self-harm occurring before the study inception date? Effectively, the study has no look-back period, which is concerning because the **length of lookback period affects incidence estimates** (e.g. <https://www.jclinepi.com/article/S0895-4356(06)00058-8/abstract>)

It was not our aim to describe individuals’ first intentional self-harm event in their lifetime, but rather the first documented event following study entry. The original titles of Figures 1 and 2, and subheadings in the Results section may have been misleading in this regard. We had chosen these titles to emphasize that our analysis does not include recurrent events, as claims data do not allow to reliably distinguish between follow-up care for the index event and true recurrent events and we could not consider recurrent events.

Introducing a lookback period of a maximum of one or two years (considering median follow-up time of 3 years) would not ensure that the first observed event reflects an individual’s first lifetime event, given that the mean age at baseline among those with documented self-harm is 29 years, while self-harm incidence peaks at around age 20. Many first events would likely still have occurred before to the lookback window.

We have revised the wording in the figure titles and Results section to avoid implying that the first documented event corresponds to an individual’s first lifetime episode. Instead, we now explicitly state in the figure captions and Methods section that recurrent events were not considered, and we discuss the implications of this limitation for incidence estimates.

For example, the revised figure titles and captions for Figures 1 and 2 now read:

**“Figure 1. Incidence of self-harm by age and sex**

Panel A presents the incidence per 100,000 person-years as a continuous function of age, for females and males. Panel B shows the Aalen-Johansen estimate of cause-specific cumulative incidence over time since baseline or age-group change, stratified by sex and age group. **Individuals were censored at the time of their first documented self-harm event. Repeated self-harm events were not considered.**

**Figure 2. Predictors of self-harm**

The figure shows age- and sex-adjusted and fully adjusted hazard ratios for predictors of self-harm. Individuals were censored at the time of their first documented self-harm event. **Repeated self-harm events were not considered**. […]"

We also clarified this point in the Methods section under “Outcome” and “Statistical analysis (self-harm analysis)”

Outcome: “In the self-harm analysis, the outcome was an individual's first observed healthcare encounter for intentional self-harm (ICD-10 codes X60-X84) [31]. **Repeated self-harm events were not considered**.”

Statistical analysis: “We estimated cause-specific cumulative incidence of self-harm using the Aalen–Johansen estimator, treating death as a competing event and stratifying by age group and sex [33]. **Individuals exited the risk set upon first self-harm.”**

We acknowledge the exclusion of recurrent self-harm episodes as a limitation in the manuscript (Discussion, second-to-last paragraph):

“This study relies on routine data, which has inherent limitations. First, intentional self-harm could be underestimated for several reasons: **we could not consider recurrent self-harm episodes**; […]”

4.      P4, paragraph 3. Can the codes for 'unnatural deaths' be included?

We have included the ICD-10 codes for unnatural deaths in the Methods section under “Outcomes”:

“In the mortality analysis, the outcome was death from unnatural causes **(ICD-10 codes V01-Y98)**, including suicides and fatal accidents.”

These codes are also included in the Abstract:

“We estimated the cause-specific cumulative incidences of healthcare encounters for intentional self-harm (ICD-10 codes X60-X84) and unnatural deaths (**ICD-10 codes V01-Y98**), using the Aalen-Johansen method.”

5.      P4, paragraph 4. Were the insurance and management program databases the source for these covariates?

The medical scheme and HIV program databases were the source of all variables except mortality data. We have clarified variables included in each of the two data sources in the Methods section under “Data sources.”

**“The dataset [medical scheme and HIV program] included sociodemographic characteristics, reimbursement claims for inpatient and outpatient care with International Classification of Diseases (ICD-10) diagnoses, pharmacy claims, and laboratory results.** These data were deterministically linked to vital registration records from the National Population Register using national identifiers […]. **Vital registration data included date of death, and cause of death (natural, unnatural, or unknown).**”

6.      P4, paragraph 5. Presumably those who died during the first healthcare encounter would have no or very little follow-up data?

Follow-up began at the earliest of the following: enrolment in the medical scheme or HIV program, the individual's 10th birthday, or January 1, 2011. Individuals who died during their first self-harm encounter did not necessarily have little or no follow-up, as they were followed from baseline until the time of death.

We have clarified the follow-up in the Methods section under the “Statistical analysis”.

“Baseline was defined as the latest of medical scheme or HIV program enrollment, the individual's 10th birthday, or January 1, 2011. […] [In the mortality analysis], individuals were followed from baseline until the earliest of end of insurance coverage, database closure, or death.”

7.      Was non-fatal self-harm exposure included a fixed covariate, or was this a time-varying covariate?

Time-varying variables were modelled as time-varying covariates.

We clarified this in the Methods section under “Statistical analysis” in the first paragraph.

“Age group, non-fatal self-harm, HIV status, and mental disorders were modelled as time-varying covariates, with individuals considered exposed from the first recorded occurrence.”

8.      Table S3. With your sample size, even a trivial difference will be 'significant', I would consider putting in an **estimate of standardised difference** instead of the p-value.

We have replaced p-values with estimates of the standardized difference in former Table S3 now Table C in Appendix S1 to provide a more meaningful assessment of group differences given the large sample size. The Methods sections has been updated accordingly.

Text A in Appendix S1. *Methodological details for post-hoc analysis*

“We calculated standardized differences to assess groups group differences, interpreting values <0.1 as not meaningful, >0.2 as moderate, and >0.5 as large [2].

**Reviewer #2**
Thank you for the opportunity to review this informative manuscript. The authors present a valuable and rare dataset from South Africa, offering insights into the risk factors for self-harm and its subsequent mortality outcomes in a non-Western setting. While the study could make potentially important contributions to the literature, particularly in under-researched contexts, I have several major concerns related to the methodological clarity, study design, analytical approaches, and combining two studies in one manuscript.

Thank you for your thoughtful review of our manuscript and for recognizing the value of our study in advancing suicide prevention efforts in South Africa, an under-researched setting with a high burden of self-harm and a substantial unmet need for evidence-based suicide prevention. We appreciate your constructive feedback, which has helped us to improve the clarity and rigor of the manuscript. We address each of your comments in detail below and have revised the manuscript accordingly.

Comments on the overall study

1.      This manuscript presents valuable data with the potential to significantly inform suicide prevention efforts in South Africa and similar contexts. However, the combination of two complex studies, unclear definitions, missing methodological details, and insufficient adjustment for key covariates limit its current clarity and rigor. I would recommend revising the manuscript to either (1) **more clearly separate and expand upon the two components**, or (2) divide it into two distinct papers with improved methodology and presentation. [Editorial note: we prefer that you do not divide the manuscript into two independent papers]

We follow the editorial team’s recommendation to present both analyses in a single comprehensive manuscript. To improve clarity and rigor, we have added methodological detail and now more clearly distinguished the two components of the analysis throughout the manuscript.

The Methods section was restructured with subheadings to separately describe the statistical approaches for the self-harm and mortality analyses. We also introduced subheadings to distinguish pre-specified, post-hoc, and sensitivity analyses. To enhance transparency and methodological rigor, we included the study protocol (Appendix S2), a list of protocol deviations (Appendix S3), the STROBE checklist (Appendix S4), and statistical code (via GitHub) as supplementary materials.

In addition, we clarified the prognostic aim of our study (see response to Reviewer 1, comment 2), explained the rationale for using two data sources, and justified the integration of the medical scheme and HIV program cohorts (see response to Reviewer 1, comment 1).

2.      This manuscript combines two conceptually distinct yet related studies: one that investigates risk factors for first-time self-harm in the general population ("self-harm study"), and another that examines the association between self-harm and subsequent unnatural death ("mortality study"). While these topics are connected, they require different study designs, assumptions, and analyses. Presenting both studies in a single paper leads to conceptual and methodological confusion. A clearer distinction in structure and explanation between these two studies is necessary, and it may be more appropriate to report them in separate papers.

We appreciate this thoughtful observation and understand the concern regarding the conceptual distinction between the two components of our study. We agree that the previous version of our manuscript may not have fully conveyed the overarching prognostic aim, which may have contributed to this impression. In the revised version, we now more clearly articulate our overall objective and explicitly distinguish the two analytical components (see our response to your previous comment), while emphasizing their shared purpose.

Both analyses are prognostic in nature and closely interrelated, addressing two sequential and complementary questions essential to self-harm/suicide prevention: first, identifying individuals at risk of self-harm to inform early detection and population-level prevention strategies; and second, evaluating the risk of unnatural death following a non-fatal self-harm event to guide indicated suicide prevention strategies targeting individuals presenting with self-harm. These aims align with WHO recommendations, which highlight early detection and indicated suicide prevention interventions as key effective evidence-based strategies [1].

Importantly, the WHO’s implementation guide recommends that countries conduct a situational analysis to collect context-specific epidemiological data prior to implementing suicide prevention interventions [1]. Our study directly contributes to this evidence base by providing data from South Africa, a high-burden and under-researched setting. We therefore believe that both analytical components offer essential and complementary insights.

We now state these points more clearly in the second-to-last paragraph of the Introduction to emphasize to conceptual coherence of the two analytic components:

“The World Health Organization (WHO) recommends early identification of individuals at risk of self-harm and suicide, and management, support, and monitoring of those with a history of self-harm as key effective suicide prevention strategies [19]. The organization urges countries to implement evidence-based strategies tailored to local contexts and informed by local data [19], to achieve the international target of reducing suicide mortality by one third from 2015 levels by 2030 [19,20]. However, current data on self-harm in South Africa are scarce, primarily derived from small, cross-sectional, single-center studies without control groups [11,12,21–23]. The existing studies have not estimated the incidence or predictors of self-harm and subsequent mortality. Such estimates are essential to support early identification of at-risk groups and to inform the development of evidence-based strategies for managing individuals presenting with self-harm, tailored to their risk of subsequent unnatural death.”

With regard to study design, both analyses are appropriately addressed within a cohort study framework using survival analysis and a shared general population reference group. Given their conceptual and methodological coherence, and in line with the editorial team’s recommendation, we believe that presenting them within a single manuscript offers a more comprehensive and clinically relevant contribution.

3.        The study excluded individuals who could not be linked to mortality records. However, no **comparison is provided between included and excluded individuals**. It is essential to assess whether exclusion may have introduced bias. Basic comparisons of self-harm incidence, sociodemographic composition, and the distribution of mental disorders in the included and excluded samples should be presented. It is unclear whether the exclusion was applied to both the self-harm study and mortality study.

Thank you for highlighting this important point. We agree that the exclusion of individuals not linked to mortality data warrants careful consideration. To clarify, individuals who could not be linked to the national mortality registry were included in the self-harm incidence analyses and only excluded from the mortality analysis. Eligibility criteria for each analysis are clearly stated in the Methods section under “Study design and participants” and is illustrated in the CONSORT-style flowchart (Figure A in Appendix S1).

“We included individuals aged 10 years or older who were covered by the medical scheme (1 Jan 2011–30 Jun 2020) or enrolled in the HIV program (1 Jan 2011–1 Oct 2022) (Figure A in Appendix S1). We excluded HIV program members also included in the medical scheme cohort, and individuals with unknown sex. **From mortality analysis, we excluded individuals not linkable to the vital registration system**.”

In response to your suggestion, we have now added a detailed sensitivity analysis comparing individuals included versus excluded from the mortality analysis. A brief summary of key differences between the two groups is provide in the Results section in the second paragraph under “Sensitivity analyses”.

“Individuals excluded from mortality analysis were younger, had slightly higher HIV prevalence, lower prevalence of depression and anxiety (Text C, Table F in Appendix S1), and a higher incidence of self-harm (aHR 1.25, 95% CI 1.15–1.36) than those included.”

More detailed results are provided in Text C in Appendix S1.

“**Text C. Detailed results from sensitivity analyses comparing individuals included and excluded from mortality analyses**

Compared with the medical scheme cohort, the HIV cohort included a higher proportion of females (59.0% vs 51.8%) and fewer individuals aged under 25 years (11.1% vs 32.3%). The proportions diagnosed with mental disorders, and the proportion who died during follow-up, were higher in the HIV program cohort. However, these estimates are not directly comparable due to differing follow-up durations (median 9.0 years in the HIV cohort vs 3.1 years in the medical scheme cohort) (Table E).

In sensitivity analyses restricted to the medical insurance cohort, findings on the incidence of self-harm, its predictors, and non-fatal self-harm as a predictor of unnatural death were consistent with the primary analysis, although strength of associations changed somewhat. Specifically, the incidence of intentional self-harm was largely unchanged: among males, the incidence remained at 90 per 100,000 person-years (95% CI 86–94); among females, it decreased slightly from 183 to 180 per 100,000 person-years (95% CI 174–185). Age-specific incidence functions (Figure D) and cumulative incidence curves (Figure E) closely resembled those observed in the primary analysis. The association between a positive HIV status and self-harm incidence was modestly stronger (HR 1.32, 95% CI 1.22–1.44) (Figure F) compared with the estimate from the primary analysis (HR 1.23 CI 1.16–1.30). Associations between mental disorders and the incidence of self-harm remained stable in the sensitivity analysis, with only minor changes in the strength of associations (Figure F). Associations between prior non-fatal self-harm and subsequent unnatural death were slightly stronger (Figure G).

Findings were not robust, however, in sensitivity analyses of self-harm method as a predictor of unnatural death. Among females, associations with highly lethal methods were stronger in the medical scheme cohort (age-adjusted HR 40.50, 95% CI 10.10–162.00; Figure H) compared with the primary analysis (HR 29.2, 95% CI 7.29–117.0). Among males, use of highly lethal methods at the index self-harm encounter was no longer a statistically significant predictor of subsequent unnatural death (age-adjusted HR 5.05, 95% CI 0.71–35.90; Figure H), due to the exclusion of several male deaths involving highly lethal methods that occurred in the HIV program.”

We reflected on its implications for generalizability in the Discussion in the 7^th^ paragraph.

“Fifth, the mortality analysis relied on linked vital registration data, and 7% of beneficiaries who could not be linked were excluded. These individuals differed from those included, which may limit generalizability. However, given that they represented a small proportion of the cohort, the impact on findings is likely minimal. Notably, the excluded group had a higher incidence of self-harm and may have been at greater risk of unnatural death, potentially biasing the association towards the null. We therefore consider our estimates conservative.”

4.      In both studies, the use of Cox regression assumes proportional hazards, yet there is no report on whether this assumption was tested. Given that mortality risk after self-harm may decline sharply over time, checking and reporting this assumption is essential.

We agree with the reviewer that the proportional hazards assumption may not hold in this context, as suicide risk is typically highest shortly after a self-harm event [6]. This temporal pattern is reflected in our cumulative incidence curves for unnatural death, which rise steeply after the index event and then flatten over time (Figure 3).

Importantly, even if the proportional hazards assumption does not hold, hazard ratios from Cox models can be interpreted as time-averaged relative risks over the follow-up period [7]. This interpretation remains valid when hazards do not cross (as is expected here) and provides a clear and interpretable summary of relative risk across groups, which is more straightforward to communicate than parametrically modelled time-varying hazard ratios [7]. We have clarified this interpretation in the Discussion in the 6ths paragraph.

“Due to non-proportional hazards, the HRs for non-fatal self-harm should be interpreted as weighted averages of time-varying effects over the follow-up period [45].”

In addition, we now present age-adjusted, time-varying hazard ratios for non-fatal self-harm as a predictor of unnatural death (Figure C, Appendix S1).

“Time-varying HRs for non-fatal self-harm as a predictor of unnatural death are shown in Figure C in Appendix S1.”

We thank the reviewer for prompting this addition.

5.      While the authors include some variables such as HIV status and psychiatric diagnoses, important sociodemographic and clinical risk factors are missing. These include physical **comorbidities and socioeconomic status**, which are known to influence both self-harm risk and mortality.

We understand the reviewer’s concern that socioeconomic status and physical comorbidities may confound the association between non-fatal self-harm and unnatural death. This is a valid consideration in the context of causal inference. However, the objective of our study is prognostic and not etiologic. In prognostic research, confounding in the traditional causal sense is less central [8].

The aim of the analysis of non-fatal self-harm as a predictor for unnatural death is to quantify the risk increase of unnatural death in individuals with a history of non-fatal self-harm compared to the appropriate general population controls. We do not posit that non-fatal self-harm causes future suicide or other unnatural death, but rather that it is a risk marker that indicates future elevated risk. This marker can be used to identify a clearly defined population in need of indicated brief suicide prevention interventions (e.g. [9–12]), in line with WHO’s LIVE LIFE framework [1]. We have conducted formative qualitative and quantitative work and applied for funding to develop and test such an intervention for South Africa. The present study forms part of our ‘situational analysis’ to provide locally relevant data on the epidemiology of self-harm to design context-sensitive interventions, as recommended in WHO’s implementation guide [1].

In our paper, we present two adjusted model: a sex-stratified, age-adjusted model, which we consider the most informative, and a fully adjusted model. The sex-stratified, age-adjusted model provides an estimate of excess risk in people with a history of non-fatal self-harm relative to those of the same age and sex without non-fatal self-harm history, without “over-adjusting” for characteristics that form the prognostic profile of the self-harm population (e.g., their much higher prevalence of mental disorders, presumably higher prevalence of physical comorbidities etc..). Given that female sex and young age are associated with higher self-harm incidence and lower suicide mortality, adjustment for age and sex is necessary to ensure a meaningful comparison.

The fully adjusted model additionally controls for mental disorders, key predictors of suicide. This model demonstrates that non-fatal self-harm remains a strong predictor of unnatural death even after adjustment for these factors, highlighting that elevated risk exists even among individuals without underlying mental disorders. These individuals may have engaged in self-harm as an impulsive act or maladaptive coping strategy. It is plausible that such individuals remain at increased risk of suicide due to repeated impulsive or maladaptive coping behavior that may escalate over time. We believe these estimates can inform clinical care for individuals who engage in self-harm in the absence of an underlying mental disorder (about 20% in our sample).

Further adjustment of factors like physical comorbidity or socioeconomic status would change the interpretation of estimates. It would imply estimating relative risk under in the scenario where these characteristics are equally distributed in people with and without non-fatal self-harm, although they form an integral part of the diagnostic prognostic profile of the self-harm population. As such, such an estimate is not of particular clinical relevance as it would apply to a hypothetical population that does not exist in the real world (i.e. self-harm cases with level of risk factors distributed as in the general population). For this reason, we believe that our current approach is appropriate, generating clinically meaningful and policy-relevant estimates to inform indicated suicide prevention interventions.

Following this rationale, we have removed HIV status from the fully adjusted model, as it forms part of the diagnostic profile. In the previous version, HIV was included to account for oversampling of people with HIV. However, given that we now present detailed sensitivity analyses to evaluate the impact of oversampling on study results, its inclusion in the primary analysis is no longer necessary and would complicate the interpretation of the adjusted estimate.

We acknowledge that the prognostic nature of our study was not clearly stated in the original version and apologize if this created confusion. We have now clarified our objectives throughout the manuscript (see our response to Reviewer 1, Comment 2) and elaborated on the interpretation of the two adjusted estimates (i.e. the sex-stratified, age-adjusted vs. the fully adjusted estimate) in the 5th paragraph of the Discussion:

“Non-fatal self-harm was a strong predictor of subsequent unnatural death in both sexes. […] We present two adjusted analyses for this predictor which should be interpreted differently based on their adjustment sets. The sex-stratified, age-adjusted analysis indicates the excess risk of unnatural death among individuals with a history of self-harm compared to general population controls of the same age and sex, without adjusting for factors such as mental disorders that form the prognostic profile of people who engage in self-harm. We consider this analysis the most informative, as it captures the overall excess mortality burden associated with self-harm, reflecting the real-world distribution of suicide risk factors faced by affected individuals. This includes a high prevalence of psychiatric illness, affecting almost 80% of our sample, along with a presumably high burden of other psychosocial risk factors, somatic illnesses, and genetic predispositions. In fully adjusted models, additionally accounting for mental disorders, non-fatal self-harm remained a strong predictor, indicating elevated mortality risk even among self-harm survivors without psychiatric conditions. These findings highlight the urgency of implementing indicated interventions, such as safety planning [45–47] and suicide-specific brief therapies [48,49], as recommended by WHO [19], for all individuals with a history of self-harm, including those without mental disorders. Immediate post-discharge care is especially critical, given the high risk of reattempt and suicide during this period [13,15].”

6.      The study treats psychiatric conditions in a relatively broad way. However, certain diagnoses—such as ADHD and conduct disorders—are more relevant in youth, whereas depression and substance use may be important in both younger and older adults. **Age-specific modelling of psychiatric risk factors** would provide more meaningful insights.

We agree that ADHD and conduct disorders are relevant conditions among younger age groups. However, modelling age-specific effects would require interaction terms or age-stratified analyses, which we were unable to perform due to limited statistical power arising from the relatively small number of self-harm events among exposed individuals.

Comments on the self-harm study:

7.      The self-harm study attempts to identify first-time self-harm events, but no information is given on how prior episodes were ruled out. A standard approach would involve applying a washout period (e.g., 1-2 years) to reduce the likelihood of misclassifying repeat events as first-time episodes. This methodological limitation should be addressed and discussed.

Please refer to our response to Reviewer 1, comment 3, where we address this point in detail.

8.      Death was treated as a competing event in the self-harm study where it should have instead been treated as a censoring event.

Death was appropriately treated as a competing event in the estimation of cumulative incidence functions for non-fatal self-harm (Figure 2B), because death precludes the occurrence of future self-harm [13]. The use of a naïve Kaplan–Meier estimator (i.e. censoring at death) would overestimate the cumulative incidence of self-harm in the presence of death as a competing event [13]. In the Cox regression analyses we estimated cause-specific hazard ratios for predictors of self-harm incidence, treating death as a censoring event, consistent with the reviewer’s suggestion. We have clarified this approach in the revised Methods section.

“We used Cox regression to estimate age- and sex-adjusted and fully adjusted hazard ratios (HRs) for predictors of self-harm, **treating death as a censoring event**.”

Comments on the mortality study:

9.      The mortality study focuses on unnatural death, a key outcome among people with a history of self-harm. However, natural causes could be also major contributors to subsequent mortality, particularly older self-harm patients. It would strengthen the paper to provide data on all-cause mortality, and then further disaggregate by natural vs. unnatural causes.

# Individuals with a history of self-harm are expected to have a higher prevalence of physical illness are known risk factors for self-harm. As such, higher mortality from natural causes is anticipated in this population, and our data confirm this pattern. Individuals with prior self-harm had a 26% (males) to 33% (females) increased risk of death from natural causes (see Table below).

# Table. Hazard ratios for non-fatal self-harm as predictor for natural death by sex

|  | **Females** | | |  | **Males** | | |
| --- | --- | --- | --- | --- | --- | --- | --- |
|  | HR | 95% CI | p-val |  | HR | 95% CI | p-val |
| **Previous non-fatal self-harm encounter^*^** |  |  |  |  |  |  |  |
| No | 1 |  |  |  | 1 |  |  |
| Yes | 1.33 | (1.06 - 1.66) | 0.013 |  | 1.26 | (0.97 - 1.64) | 0.09 |

* Previous health-care encounter for self-harm that was non-fatal, i.e. the individuals were released from care alive.

Hazard ratios non-fatal self-harm encounter were adjusted for age, HIV status, and mental disorders.

Abbreviations: HR, adjusted hazard ratio; CI, confidence interval.

We did not include these findings in the manuscript, as analyses of natural or all-cause mortality would require a separate conceptual motivation. The aim of the mortality analysis is to inform suicide prevention efforts. Natural causes of death are not preventable through suicide prevention interventions, and elevated risk may reflect higher comorbidity in this population. To maintain conceptual clarity and focus, we therefore prefer to limit our analysis to unnatural deaths.

10.     The authors should report the proportion of deaths with unknown (natural vs unnatural causes) or undetermined (suicide vs accident) causes and discuss their implications.

These proportions are reported for the whole study population in Table 1 and for the self-harm population in Table 2.

11.     Age is a critical modifier in both the risk of self-harm and its mortality consequences. Yet, the study does not **present age interactions** or age-stratified analyses. Furthermore, while some subgroup analyses were performed, such as by method lethality and sex, no **formal tests of interaction** were conducted to assess the statistical evidence of effect modifications.

We agree that age is a critical factor in self-harm risk and present detailed age-specific incidence analyses (Figures 1 and 2). While age interactions with other predictors are of academic interest, they are difficult to interpret and communicate clearly. Given our focus on providing local data to inform suicide prevention strategies and help healthcare providers identify at-risk populations, we believe that complex regression outputs involving interaction terms offer limited practical value in this context. We plan to develop prediction models for both outcomes in future work, where interaction terms can be appropriately incorporated to generate individual-level risk scores for clinical use.

12.     In the mortality study, self-harm is modelled as a fixed exposure. However, self-harm occurs at varying times during follow-up and should be treated as a time-varying covariate in survival analyses.

All time-varying variables were modelled as time-varying covariates. We have clarified the modelling of time-varying covariates used in the mortality study in the first paragraph under “Statistical Analysis” in the Methods section.

“Age group, non-fatal self-harm, HIV status, and mental disorders were modelled as time-varying covariates, with individuals considered exposed from the first recorded occurrence.”

12. (continued) Alternatively, a matched cohort design could strengthen causal inferences.

We apologies if we did not make it sufficiently clear in the previous version of the manuscript that causal inference was not our aim. We have now clarified throughout the revised that our objective was prognostic.

12. (continued) However, the control selection is under-explained. For instance, the index date for controls should correspond to the event date of the matched self-harm case, but this is not clearly stated. Furthermore, no information was given regarding how this matching was implemented. Matching variables included age group (e.g., 40-59 years), but such broad categories can introduce residual confounding. Narrower bands (e.g., 5-year groups) should be considered. The inclusion of sex in the matching process would be also relevant and indicated.

We understand that the reviewer is referring to the analyses of “Healthcare utilization before self-harm events compared with matched controls prior to hypothetical matched event dates” (now Table C in Appendix S1).

Matching methods are explained in detail in Text A of Appendix S1:

“Finally, we used descriptive statistics to compare healthcare utilization prior to self-harm encounters with that of matched controls prior to hypothetical encounter dates. For this analysis, each self-harm case was matched to two randomly selected controls who had no self-harm encounter. Matching criteria included the same baseline year, age group (10-14, 15-19, 20-39, 40-59, 60+), and duration of follow-up (<2 years, 2-4 years, 4-6 years, 6-8 years, or 8+ years). The hypothetical event date for controls was set as their baseline date plus the interval from baseline to the self-harm encounter of the matched case. “

The mean age of the self-harm population and controls was similar (28.7 vs 29.6 years), suggesting minimal residual confounding unlikely to meaningfully affect the results of this descriptive and exploratory analysis. No matching was applied in other analyses.

13.     The final part of the paper describes post-self-harm healthcare contact within specific time frames (e.g., 1 week, 1 month). This is an important but distinct topic from the core analyses, and would be better suited to a separate, focused manuscript.

This analysis of healthcare utilization before self-harm is highly relevant to our aim of informing prevention strategies and closely linked to the self-harm analysis. It highlights opportunities for timely intervention, as most individuals accessed outpatient care shortly before the self-harm event. The utilization analysis therefore complements the self-harm predictor analysis by identifying where screening could occur, while the latter informs who should be targeted.

**Reviewer #3**
Thank you for the opportunity to review this manuscript on a topic of clear importance. The methods are quite sound and the findings are striking and far-reaching, with potential for major impact in the field. I appreciated the **extremely sounds methods and statistical approach** and **concise presentation** of a variety of findings, but I believe the manuscript would benefit from some clarifications and added detail in a few areas, outlined below.

Thank you for your thoughtful review and for recognizing the importance of our work. We appreciate your positive assessment of the methodological and statistical approach, as well as your constructive suggestions for improving the clarity and detail of the manuscript. We address each comment in detail below.

1.      Abstract: The focus on unnatural death (rather than death by suicide) is logical and the ICD codes used to define unnatural death are appropriate. However, a definition of what constitutes unnatural death would strengthen the abstract.

We have added a brief definition of unnatural death in the Background section of the Abstract:

“Individuals with a history of self-harm are at increased risk of unnatural death**, including suicide and fatal accidents**.”

2.      I appreciated the acknowledgement of some of the key limitations in the abstract.

Thank you for your comment.

3.      Introduction: In paragraphs two and three, "Non-fatal suicidal behavior" seems to be used interchangeably with "suicide attempts", which is not the case. Please address.

Thank you for pointing this out. We have completely rewritten the Introduction and now frame the study within the literature on intentional self-harm. This revised framing avoids conflating non-fatal suicidal behavior with suicide attempts and ensures terminological consistency. Please also refer to our response to Academic Editor, Comment 1.

4.      HIV status is considered in the methods and analytic plan but is completely absent from the abstract and introduction. In the prior literature, there is a link between living with HIV and suicide risk. This should be acknowledged and addressed.

We have added a sentence to the abstract to indicate that HIV-positive status was a modest predictor of self-harm.

“Mental health and substance use disorders were strong risk factor for self-harm**, while a positive HIV status was a modest predictor.**”

We have added a sentence to the 6^th^ paragraph of the Discussion to speak to suicide risk in people with HIV. We believe this is best discussed in the Discussion section, as relevant literature from South Africa is extremely limited, and findings from high-income countries, where HIV predominantly affects key populations such as men who have sex with men and people who inject drugs who are per-se at increased suicide risk, is not generalizable to the South African context of a generalized HIV epidemic.

“Predictors of self-harm are also generally consistent across public- and private-sector settings. In a study conducted in public primary care clinics in deprived neighborhoods in Cape Town, we found high rates of recurrent suicidal behavior—supporting our finding that non-fatal self-harm is an important risk marker—and strong associations between self-harm, female sex, and mental disorders [50]. **Notably, HIV was not associated with self-harm in this study, and similarly, our current analysis found only a weak association, in contrast to findings from high-income countries where HIV is associated with substantially increased suicide risk [51].**”

5.      Add considerations of how the sample of people who had health insurance coverage might differ from the general population.

We provide additional details in the Methods section (subheading Setting) on how individuals with private medical insurance differ from the general population in terms of socio-economic status, and exposure to psychosocial stressors.

“Medical scheme beneficiaries generally have higher socio-economic status and may face fewer psychosocial stressors of self-harm than individuals without insurance [29].”

6.      Considerations of **social determinants** of health (e.g., food insecurity, housing insecurity, race/ethnicity, socioeconomic status) as predictors of unnatural death in this sample are **completely absent**. Why weren't these considered, and might it be beneficial to explore them in future studies?

Adjusting for social determinants of health may constitute overadjustment in the context of our study, where the goal is to identify high-risk individuals rather than making causal inference (see our response to Reviewer 2, Comment 5).

Furthermore, we would like to highlight that the study cohort consists of privately insured individuals, where severe deprivation (i.e. food insecurity, housing insecurity, poverty) is uncommon. In addition, such variables are not captured in the claims data.

We are highly interested in the role of social determinants in suicide risk, particularly how the confluence of psychosocial stressors related to social deprivation and interpersonal adversity contributes to suicidal behavior. We have explored these issues in a cohort study conducted in two township communities in Cape Town, that are characterized by high levels of poverty, violence, trauma, unemployment, and substance use.

We have expanded the Discussion (6^th^ paragraph) to include a paragraph reflecting on how differences regarding socioeconomic status and access to healthcare between populations accessing public vs private healthcare affect the generalizability of our findings.

“Our study population included privately insured individuals with higher socioeconomic status and better access to healthcare than the uninsured population relying on South Africa’s public sector, who may face greater psychosocial stressors [29]. While self-harm and suicide incidence may be expected to be higher in the public sector, South African evidence suggests that socioeconomic status indicators such as low education are not consistently associated with suicidal behavior [8], supporting the generalizability of our findings. Predictors of self-harm are also generally consistent across public- and private-sector settings. In a study conducted in public primary care clinics in deprived neighborhoods in Cape Town, we found high rates of recurrent suicidal behavior—supporting our finding that non-fatal self-harm is an important risk marker—and strong associations between self-harm, female sex, and mental disorders [50]. Notably, HIV was not associated with self-harm in this study, and similarly, our current analysis found only a weak association, in contrast to findings from high-income countries where HIV is associated with substantially increased suicide risk [51].”

7.      It appears the second sub-sample were people living with HIV. Given the prior literature highlighting HIV status as a risk factor for suicide, the implications of using this sub-sample should be addressed more thoroughly. Tables S5 and S6 show higher risk among people with HIV but this is not addressed in the Abstract or Results - please add.

The reviewer is referring two tables that were included in the previous version of the manuscript, which reported hazard ratios for factors associated with unnatural death among female (Tables S5) and male (Table S6) beneficiaries. As noted by Reviewer 1, these tables may be misleading, as HIV status and mental disorders were included as adjustment variables rather than exposures of interest. The associations presented reflect estimates in the overall population rather than among individuals with non-fatal self-harm and cannot be interpreted as predictors for unnatural death within the self-harm population. These tables were included in the appendix in the previous version of the manuscript for transparency. Given the potential for misinterpretation and limited relevance to the study's objective, we have removed these tables from the revise version. Results for our primary exposure (non-fatal self-harm) are presented in Figure 4A.

8.      Did the prevalence of HIV in the final sample reflect the prevalence in the general population? If people living with HIV were over-sampled, how might this have impacted the generalizability of findings?

We appreciate the reviewer’s suggestion to examine the potential influence of including the HIV program cohort. We have addressed this through several sensitivity analyses and discuss the implications in the Discussion section. For a detailed explanation, please refer to our response to Comment 3 from the Academic Editor.

9.      Similarly, comparison of baseline prevalence of common mental disorders in your sample with the prevalence in the general population will give some indication of generalizability.

The most recent nationally representative estimates for common mental disorders in South Africa are from 2002–2004 based on a population-based household survey in which all participants received structured psychiatric diagnostic interviews with high sensitivity and specificity [14]. Reported lifetime prevalence was 16% for anxiety disorders and 10% for major depressive disorder.

These research-based estimates are not directly comparable to prevalence estimates based on routine care data, where cases may remain undiagnosed. Differences in the observation periods further limit comparability, as the survey measured lifetime and 12-months prevalence, whereas our study captured routine diagnoses recorded over a median follow-up of three years. In our sample, 16% had a recorded diagnosis of an anxiety disorder and 13% of depression. These estimates are broadly consistent with population-level expectations.

Given the methodological differences between research-based and claims-based mental health diagnoses, we believe that a direct comparison would be of limited interpretability. Therefore, we have not included such a comparison in the manuscript as a basis for assessing the generalizability of our findings.

**General editorial requests**

*	We ask every co-author listed on the manuscript to fill in a contributing author statement, making sure to declare all competing interests. If any of the co-authors have not filled in the statement, we will remind them to do so when the paper is revised. If all statements are not completed in a timely fashion this could hold up the re-review process. If new competing interests are declared later in the revision process, this may also hold up the submission. Should there be a problem getting one of your co-authors to fill in a statement we will be in contact. Please do not add or remove authors without first discussing this with the handling editor. You can see our competing interests policy here: <https://journals.plos.org/plosmedicine/s/competing-interests>

The authors declare no conflicts of interest.

*	Please upload any **figures** associated with your paper as individual **TIF or EPS** files with **300dpi resolution** at resubmission; please read our figure guidelines for more information on our requirements: https://journals.plos.org/plosmedicine/s/figures. PACE helps ensure that figures meet PLOS requirements. To use PACE, you must first register as a user. Then, login and navigate to the UPLOAD tab, where you will find detailed instructions on how to use the tool. If you encounter any issues or have any questions when using PACE, please email us at [PLOSMedicine@plos.org](mailto:PLOSMedicine@plos.org).

We have uploaded figures as high-resolution TIFF as requested.

*	Please ensure that the paper adheres to the PLOS Data Availability Policy (see https://journals.plos.org/plosmedicine/s/data-availability), which requires that all data underlying the study's findings be provided in a repository or as Supporting Information. For data residing with a third party, authors are required to provide instructions with contact information (web or email address) for obtaining the data. Please note that a study author cannot be the contact person for the data. PLOS journals do not allow statements supported by "data not shown" or "unpublished results." For such statements, authors must provide supporting data or cite public sources that include it.

The authors are fully supportive of PLOS’s open science strategy. The study protocol and analytical code will be made publicly available upon publication. However, the underlying data cannot be shared publicly due to legal and ethical restrictions. The data were obtained from the International epidemiology Databases to Evaluate AIDS–Southern Africa (IeDEA-SA). To request access to the data, interested researchers may contact IeDEA-SA by completing the online request form available at: <https://www.iedea-sa.org/contact-us/>.

*	We expect all researchers with submissions to PLOS in which author-generated code underpins the findings in the manuscript to make all author-generated code available without restrictions upon publication of the work. In cases where code is central to the manuscript, we may require the code to be made available as a condition of publication. Authors are responsible for ensuring that the code is reusable and well documented. Please make any custom code available, either as part of your data deposition or as a supplementary file. Please add a sentence to your data availability statement regarding any code used in the study, e.g. "The code used in the analysis is available from Github [URL] and archived in Zenodo [DOI link]" Please review our guidelines at https://journals.plos.org/plosmedicine/s/materials-software-and-code-sharing. Because Github depositions can be readily changed or deleted, we encourage you to make a permanent DOI'd copy (e.g. in Zenodo) and provide the URL.

We have published the statistical code on GitHub, and the link is provided in the Data Availability Statement. Upon acceptance of the manuscript, we will archive the code in Zenodo and add the DOI during the proof stage. We have added the following sentence to the Data Availability Statement:

" The code used for the analysis is available on Github (https://github.com/AndreasDHaas/Self-harm-epidemiology/) and archived in Zenodo [DOI placeholder].”

FORMATTING - GENERAL

*	Abstract: Please structure your abstract using the PLOS Medicine headings (Background, Methods and Findings, Conclusions). Please combine the Methods and Findings sections into one section.

The abstract adheres to the journal’s guidelines.

*	At this stage, we ask that you include a short, non-technical Author Summary of your research to make findings accessible to a wide audience that includes both scientists and non-scientists. The Author Summary should immediately follow the Abstract in your revised manuscript. This text is subject to editorial change and should be distinct from the scientific abstract. Ideally each sub-heading should contain 2-3 single sentence, concise bullet points containing the most salient points from your study. In the final bullet point of 'What Do These Findings Mean?', please include the main limitations of the study in non-technical language. Please see our author guidelines for more information: https://journals.plos.org/plosmedicine/s/revising-your-manuscript#loc-author-summary.

We have added the Author Summary after the abstract, as requested.

*	Please express the main results with 95% CIs as well as p values. When reporting p values please report as p<0.001 and where higher as the exact p value p=0.002, for example. Throughout, suggest reporting statistical information as follows to improve clarity for the reader "22% (95% CI [13%,28%]; p</=)". Please be sure to define all numerical values at first use.

We report 95% confidence intervals for all main results. As noted by the statistical reviewer, p-values are not particularly informative in a study of this size (1.35 million individuals), as even trivial, non-clinically relevant differences may appear statistically significant. In line with the reviewer’s recommendation, we have replaced p-values in Table C in Appendix S1 with standardized differences and prefer not to report p-values throughout the study, instead relying on confidence intervals as recommended by Rothman and others [15,16].

*	Please include page numbers and line numbers in the manuscript file. Use continuous line numbers (do not restart the numbering on each page).

We have added page numbers and continuous line numbers throughout the manuscript, as requested.

*	Please cite the reference numbers in square brackets. Citations should precede punctuation.

We have followed the journal’s citation style by using square brackets for reference numbers and placing citations before punctuation.

FIGURES AND TABLES

*	Please provide titles and legends for all figures and tables (including those in Supporting Information files).

We have provided titles and legends for all figures and tables, including those in the Supporting Information.

*	Please define all abbreviations used in each figure/table (including those in Supporting Information files).

All abbreviations used in each figure and table have been defined accordingly.

*	Please consider avoiding the use of red and green in order to make your figure more accessible to those with color blindness.

We have revised the color scheme to ensure it is colorblind-friendly.

SUPPLEMENTARY MATERIAL

*	Please note that supplementary material will be posted as supplied by the authors. Therefore, please amend it according to the relevant comments outlined here.

We have revised the supplementary material in line with the comments provided.

*	Please cite your Supporting Information as outlined here: https://journals.plos.org/plosmedicine/s/supporting-information

We have cited all supplementary materials in accordance with the journal’s style.

REFERENCES

*	PLOS uses the numbered citation (citation-sequence) method and first six authors, et al.

*	Please ensure that journal name abbreviations match those found in the National Center for Biotechnology Information (NCBI) databases (https://www.ncbi.nlm.nih.gov/nlmcatalog/journals), and are appropriately formatted and capitalised.

*	Where website addresses are cited, please include the complete URL and specify the date of access (e.g. [accessed: 12/06/2023]).

*	Please also see <https://journals.plos.org/plosmedicine/s/submission-guidelines#loc-references> for further details on reference formatting.

We have followed the journal’s citation style.

OBSERVATIONAL STUDIES

*	Abstract: Please include the study design, population and setting, number of participants, years during which the study took place (enrollment and follow up), length of follow up, and main outcome measures.

The revised abstract includes the required elements.

*	Please ensure that the study is reported according to the STROBE (or appropriate STOBE extension) guideline (available from: https://www.equator-network.org/reporting-guidelines/strobe/) and include the completed STROBE (or STROBE extension) checklist as Supporting Information. Please add the following statement, or similar, to the Methods: "This study is reported as per the Strengthening the Reporting of Observational Studies in Epidemiology (STROBE) guideline (S1 Checklist)." When completing the checklist, please use section and paragraph numbers, rather than page numbers.

We included a statement noting that the study was reported in accordance with the STROBE guidelines and submitted the STROBE checklist as Appendix S4.

This study was report is accordance with STROBE guidelines (Appendix S4).

*	For all observational studies, in the manuscript text, please indicate: (1) the specific hypotheses you intended to test, (2) the analytical methods by which you planned to test them, (3) the analyses you actually performed, and (4) when reported analyses differ from those that were planned, transparent explanations for differences that affect the reliability of the study's results. If a reported analysis was performed based on an interesting but unanticipated pattern in the data, please be clear that the analysis was data driven.

Analyses were conducted according to a pre-specified protocol (Appendix S2) and protocol deviations are listed in Appendix S3. We included a statement highlighting this under Methods “Study design and participants”.

“We conducted analyses according to a pre-specified protocol (Appendix S2), list protocol deviations in Appendix S3, and […]”

We introduced subheadings in the “Statistical Analyses” section of the Methods to clearly distinguish between pre-specified and post-hoc analyses.

“Post-hoc analyses

We conducted the following additional post-hoc analyses: First, we assessed whether the lethality of the method used at the index self-harm event predicted subsequent unnatural death. Second, we used flexible parametric survival models to estimate age-adjusted, time-varying HRs for non-fatal intentional self-harm. Finally, to explore opportunities for self-harm prevention, we compared healthcare utilization before self-harm encounters with matched controls. Further details are provided in Text A in Appendix S1.

Sensitivity analyses

In post-hoc sensitivity analyses, we repeated all primary analyses in the medical scheme cohort only, to assess the impact of oversampling individuals with HIV due to the inclusion of HIV program data. To assess generalizability to individuals not linked to the vital registration system, we compared those included versus excluded from the mortality analysis. Self-harm incidence was compared using Cox regression adjusted for age, sex, HIV status, and mental disorders.”

*	Please state in the Methods section whether the study had a prospective protocol or analysis plan. If a prospective analysis plan (from your funding proposal, IRB or other ethics committee submission, study protocol, or other planning document written before analyzing the data) was used in designing the study, please include the relevant document(s) with your revised manuscript as a Supporting Information file to be published alongside your study and cite it in the Methods section. A legend for this file should be included at the end of your manuscript. If no such document exists, please make sure that the Methods section transparently describes when analyses were planned, and when/why any data-driven changes to analyses took place. Changes in the analysis, including those made in response to peer review comments, should be identified as such in the Methods section of the paper, with rationale.

Analyses were conducted according to a pre-specified protocol (submitted as Appendix S2). We state this under Methods “Study design and participants”.

“We conducted analyses according to a pre-specified protocol (Appendix S2), list protocol deviations in Appendix S3 […]”

Technical Requests - Journal Requirements:

1) Please send a completed 'Competing Interests' statement, including any COIs declared by your co-authors. If you have no competing interests to declare, please state "The authors have declared that no competing interests exist".

We included the following statement under Declarations:

“Declaration of competing interest

The authors declare no conflicts of interest.”

2) Please note that the contact for accessing your data cannot be an author on the manuscript. Please therefore replace the current contact information with a third party contact, such as an institutional data access committee.

In our Data Availability Statement, we note that “To request data, readers may contact IeDEA-SA for consideration by filling out the online form available at https://www.iedea-sa.org/contact-us/.” Data requests are received by Dr Per von Groote, IeDEA Program Manager (not an author of this study), and reviewed by the IeDEA Steering Committee in accordance with its “Principles of Collaboration” and standardized procedures (https://www.iedea-sa.org/collaborate-with-us/). To our knowledge, no separate institutional data access committee exists at our institutions.

3) We ask that a manuscript source file is provided at Revision. Please upload your manuscript file as a .doc, .docx, .rtf or .tex.

The revised manuscript was submitted as a .docx file, in accordance with the journal’s requirements.

4) Please provide separate figure files in .tif or .eps format.

For more information about figure files please see our guidelines:

<https://journals.plos.org/plosmedicine/s/figures>

<https://journals.plos.org/plosmedicine/s/figures#loc-file-requirements>

We confirm that all figures have been provided as separate high quality .tif files, as requested.

5) Please upload a copy of Figure [1-4] which you refer to in your text on your Manuscript. Or, if the figure is no longer to be included as part of the submission please remove all reference to it within the text.

We confirm that Figures 1-4 have been uploaded and are referenced in the text.

6) We notice that your supplementary [figures and tables] are included in the manuscript file. Please remove them and upload them with the file type 'Supporting Information'. Please ensure that each Supporting Information file has a legend listed in the manuscript after the references list.

We confirm that all supplementary figures and tables have been removed from the main manuscript file and submitted as a separate file (Appendix S1). Legends for each figure and table are included in the manuscript after the reference list, as requested.

**References**

1.	World Health Organization. LIVE LIFE: An implementation guide for suicide prevention in countries. Geneva: WHO; 2021.

2.	Bradshaw D, Joubert J, Maqungo M, Nannan N, Funani N, Laubscher R, et al. South African National Cause-of-Death Validation Project: Methodology and Description of a National Sample of Verbal Autopsies. 2020. Available: https://www.samrc.ac.za/sites/default/files/attachments/2022-08/NationalCause-of-deathValidationReport.pdf

3.	Dorrington R, Bradshaw D, Laubscher R, Nannan N. Rapid Mortality Surveillance Report 2019 & 2020. 2021 Nov. Available: https://www.samrc.ac.za/sites/default/files/attachments/2022-08/Rapid%20Mortality%20Surveillance%20Report%202019%262020.pdf

4.	Kraemer HC. Coming to Terms With the Terms of Risk. Arch Gen Psychiatry. 1997;54: 337. doi:10.1001/archpsyc.1997.01830160065009

5.	van Diepen M, Ramspek CL, Jager KJ, Zoccali C, Dekker FW. Prediction versus aetiology: common pitfalls and how to avoid them. Nephrology Dialysis Transplantation. 2017;32: ii1–ii5. doi:10.1093/ndt/gfw459

6.	Demesmaeker A, Chazard E, Hoang A, Vaiva G, Amad A. Suicide mortality after a nonfatal suicide attempt: A systematic review and meta-analysis. Aust N Z J Psychiatry. 2022;56: 603–616. doi:10.1177/00048674211043455

7.	Stensrud MJ, Hernán MA. Why Test for Proportional Hazards? JAMA. 2020;323: 1401. doi:10.1001/jama.2020.1267

8.	Kent P, Cancelliere C, Boyle E, Cassidy JD, Kongsted A. A conceptual framework for prognostic research. BMC Med Res Methodol. 2020;20: 172. doi:10.1186/s12874-020-01050-7

9.	Stanley B, Brown GK. Safety Planning Intervention: A Brief Intervention to Mitigate Suicide Risk. Cogn Behav Pract. 2012;19: 256–264. doi:10.1016/j.cbpra.2011.01.001

10.	Bryan CJ, Mintz J, Clemans TA, Leeson B, Burch TS, Williams SR, et al. Effect of crisis response planning vs. contracts for safety on suicide risk in U.S. Army Soldiers: A randomized clinical trial. J Affect Disord. 2017;212: 64–72. doi:10.1016/j.jad.2017.01.028

11.	Stanley B, Brodsky B, Monahan M. Brief and Ultra-Brief Suicide-Specific Interventions. Focus (Madison). 2023;21: 129–136. doi:10.1176/appi.focus.20220083

12.	Gysin-Maillart A, Schwab S, Soravia L, Megert M, Michel K. A Novel Brief Therapy for Patients Who Attempt Suicide: A 24-months Follow-Up Randomized Controlled Study of the Attempted Suicide Short Intervention Program (ASSIP). PLoS Med. 2016;13: e1001968. doi:10.1371/journal.pmed.1001968

13.	Gooley TA, Leisenring W, Crowley J, Storer BE. Estimation of failure probabilities in the presence of competing risks: new representations of old estimators. Stat Med. 1999;18: 695–706.

14.	Herman AA, Stein DJ, Seedat S, Heeringa SG, Moomal H, Williams DR. The South African Stress and Health (SASH) study: 12- month and lifetime prevalence of common mental disorders. South African Medical Journal. 2009;99: 339–344.

15.	Rothman KJ. Six Persistent Research Misconceptions. J Gen Intern Med. 2014;29: 1060–1064. doi:10.1007/s11606-013-2755-z

16.	Greenland S, Senn SJ, Rothman KJ, Carlin JB, Poole C, Goodman SN, et al. Statistical tests, P values, confidence intervals, and power: a guide to misinterpretations. Eur J Epidemiol. 2016;31: 337–350. doi:10.1007/s10654-016-0149-3
