# Supplementary material for: Incidence and prognostic factors of self-harm and subsequent unnatural death in South Africa: A cohort study
Source: PLoS Med. 2025 Sep 26;22(9):e1004765. doi: 10.1371/journal.pmed.1004765 (PMC12503312; doi:10.1371/journal.pmed.1004765)
Supplement: Attachment — Submitted filename: Responses_to_reviewers_v1.3.docx [file pmed.1004765.s007.docx]

Response to Editors’ and Reviewers’ Comments

We thank the editors and reviewers for their thoughtful evaluation of our revised manuscript "Self-Harm and Unnatural Death in South Africa: A Cohort Study" (PMEDICINE-D-25-00406R2). Below, we respond to each of the remaining comments in detail. Our responses are shown in blue; quotations from the revised manuscript are indented. Revisions to the manuscript text are highlighted in yellow, and revisions to numeric results are highlighted in green.

Thank you again for your careful review. We look forward to your feedback on the revised submission.

Sincerely,

Andreas Haas (on behalf of all co-authors)

-----------------------------------------------------------
Comments from the associate editor:

Many thanks for submitting your manuscript "Self-Harm and Unnatural Death in South Africa: A Cohort Study" (PMEDICINE-D-25-00406R2) to PLOS Medicine. The paper has been re-reviewed by the subject experts and a statistician; their comments are included below and can also be accessed here:

As you will see, the reviewers think the manuscript has improved, but have some remaining concerns. After discussing the paper with the editorial team and an academic editor with relevant expertise, I'm pleased to invite you to revise the paper in response to these last reviewers' comments. Specifically, R#1 comments on the revision being positioned as prognostic, whereas the results are still appropriate for a causality study. R#2 would like you to test for sex-differences, and if these are not present this reviewer suggests to combine the estimates. Please note that we do not expect you to address the comments regarding reporting all-cause mortality numbers as well. We plan to send the revised paper to some or all of the original reviewers, and we cannot provide any guarantees at this stage regarding publication.

Dear Dr De Bruijn,

thank you for the careful review of our revised manuscript and the opportunity to revise the paper. We respond to the specific comments from Reviewer #1 and Reviewer #2 below.

-----------------------------------------------------------
Comments from the academic editor:

1. The recommendations regarding the sex differences are valid, and should be addressed.

We respond to this comment below (see Reviewer #2, comment 2).

2. The suggestion of reporting all-cause mortality separately to unnatural deaths is beyond the scope of this paper.

We agree that this analysis is beyond the scope of the current paper.

-----------------------------------------------------------
Comments from the reviewers:

**Reviewer #1**:

Thanks for the revised manuscript and responses to my original review. The updates have clarified most of my original queries.

Thank you for your careful review of our revised manuscript. We are pleased that the revisions have clarified most of your original queries. We respond to the remaining concerns below.

1. My remaining concern is that the updates to the manuscript now position the aim of the study to be prognostic, rather than to estimate a causal relationship between the different study covariates and risk of self-harm and unnatural deaths. However, the methods described and the way the results are reported are for an aetiological study rather than a prediction study. If the aim was a predictive model of the study outcomes, the study needs a) a measure of discrimination of the model (e.g. c-statistic), b) level of calibration of predictions from the model, c) a way of measuring or managing model optimism (cross-validation, bootstrapping), and d) a clear vision of the 'use case' of the model, i.e. where it could be implemented and what actions could be taken with predictions from the model are available. The individual coefficients from the model should not be interpreted as effect estimates either (e.g. see Box 2 of van Diepen 2017 which was referenced in the response). The manuscript should report appropriate results of whatever approach is chosen as the aim of the study (descriptive, predictive, causal, see this recent reference summarises this well: https://onlinelibrary.wiley.com/doi/10.1002/sim.10244

We appreciate the opportunity to clarify the type of prognostic research our study represents and the appropriate reporting standards for this type of study. According to the PROGnosis RESearch Strategy (PROGRESS) framework [1], there are four types of prognostic research: (1) fundamental prognostic research, (2) prognostic factor research, (3) prognostic model research, and (4) stratified medicine research. Prognostic factor research investigates prognostic factors, defined as “any measure that, among people with a given startpoint (such as diagnosis of disease), is associated with a subsequent endpoint (such as death).” [2], whereas prognostic model research aims to develop and validate statistical models to predict individual risk of a future outcome [3].

Because we describe the study as prognostic, the reviewer seems to assume it is prognostic model research (PROGRESS category 3) and therefore recommends TRIPOD-compliant reporting, including measures of discrimination and calibration [4]. However, we conducted prognostic factor research [2] (PROGRESS category 2), which has different objectives and reporting standards from prognostic model research [3]. We investigate whether characteristics such as sex, age group, mental disorders and HIV are associated with future self-harm (self-harm analysis) and whether non-fatal self-harm is associated with subsequent unnatural death (mortality analysis). In both analyses, we follow individuals from a clearly defined start point, namely enrolment in the medical scheme or HIV program, after which all individuals are at risk of experiencing the outcome of interest. Our study’s objectives and design align with prognostic factor research, and it is reported accordingly [2,5].

The reviewer’s statement that individual model coefficients should not be interpreted is correct for prognostic model research, where variables are selected for predictive performance and individual coefficients are not the primary output. In such studies, discrimination and calibration measures are essential, and TRIPOD reporting applies. By contrast, in our prognostic factor study, variables were included based on clinical and epidemiological knowledge and were not removed due to lack of association or limited predictive performance. Evaluating whether a certain prognostic factor remains associated with the outcome after adjustment for other known predictors is an encouraged practice in prognostic factor research (see for example Figure S1 in PROGRESS 2 or items 16 and 17 in the REMARK checklist [2,5]). We thus consider the interpretation of individual coefficients appropriate and informative for our study objectives.

We interpret the estimates from our study as prognostic associations and not as causal effects, as unmeasured confounding is likely and we did not apply causal inference methods. Since our aim is not to identify intervention targets (for example, treating a specific mental disorder to reduce self-harm risk) but to identify groups at increased risk (for example, female adolescents who may benefit from targeted interventions), establishing prognostic associations is sufficient and causal inference is not required.

To clarify these points in the manuscript, we have made several revisions to explicitly position our study as an example of prognostic factor research as defined in the PROGRESS framework. These changes include edits to the introduction, methods, and discussion, as outlined below.

In the last paragraph of the introduction, where we state the aims of our study, we removed the vague phrase “prognostic in nature” and replaced the reference to van Diepen et al. (2017), which relates to prognostic model research, with Riley et al. (2013), which relates to prognostic factor research. Van Diepen et al. had been cited to support the point that prognostic research does not necessarily imply causality. However, we recognized that citing a reference related to prognostic model research created confusion in the context of a prognostic factor study.

“To address these gaps, we analyzed longitudinal data from over one million beneficiaries of South African medical insurance schemes. We aimed to estimate self-harm incidence, identify its predictors, and to evaluate non-fatal self-harm as a predictor of subsequent unnatural death. Our analyses were ~~prognostic in nature and~~ not designed to identify causal (etiologic) risk factors. Instead, we aimed to identify individual-level characteristics that serve as risk markers, indicating increased future self-harm or mortality risk, without necessarily implying causality [24].”

In the *Study design and participants* section of the Methods, we clarified that our study aligns with prognostic factor research, as defined in the PROGRESS framework. We also explained that we use the term “predictor” instead of “prognostic factor” as we study a general population of medical insurance beneficiaries, not individuals with a specific disease.

“*Study design and participants*

We conducted a cohort study using longitudinal reimbursement claims data from a South African medical insurance scheme (hereafter referred to as the medical scheme) and a private sector HIV disease management program (hereafter referred to as the HIV program). The design and objectives of this study align with prognostic factor research [24], as defined in the PROGnosis RESearch Strategy (PROGRESS) framework [25]. We refer to prognostic factors as ‘predictors’ throughout the manuscript, as we study a general population of medical insurance beneficiaries, not individuals with a specific disease.”

We incorporated the requirement “among people with a given startpoint” from the definition of prognostic factors into our definition of predictors and added the reference to Riley et al 2013 (PROGRESS 2).

“*Predictors*

We defined predictors as individual-level characteristics, measured before the outcome among people with a given start point, that indicate increased risk of future self-harm or mortality, without necessarily implying causality [24,32].”

**Reviewer #2:**

I thank the authors for their responses. I have two further comments on the revised paper as outlined below:

1. The authors argued that "The aim of the mortality analysis is to inform suicide prevention efforts. Natural causes of death are not preventable through suicide prevention interventions, and elevated risk may reflect higher comorbidity in this population. To maintain conceptual clarity and focus, we therefore prefer to limit our analysis to unnatural deaths." However, suicide prevention activities that incorporate psychosocial support may have spillover effects on overall health, including physical health. Moreover, a holistic approach to the care of individuals who have self-harmed should address both mental and physical health needs. Importantly, all-cause mortality is a key overarching outcome for this population, and estimating its risk can help contextualise the relative contributions of natural and unnatural causes of death. Therefore, I wonder if the authors could consider providing risk estimates for all-cause and natural-cause mortality, at least in the supplementary materials, if not in the main text, to offer a more comprehensive understanding of mortality risk in this population.

We thank the reviewer for this thoughtful suggestion and fully agree that a holistic approach to the care of individuals who have self-harmed should consider both mental and physical health outcomes. We also agree that suicide prevention interventions may have broader benefits beyond reducing deaths from unnatural causes.

However, in line with the academic editor’s assessment, we believe that a detailed analysis of all-cause and natural-cause mortality is beyond the scope of the current paper. Our focus is on informing suicide prevention efforts by identifying risk factors for self-harm and unnatural death. Including a parallel analysis of all-cause mortality would require substantial additional conceptual and methodological considerations and risks diverting focus from the main aim of the study.

2. In Figure 4, sex-specific analyses were presented. However, the 95% confidence intervals for hazard ratios in males and females overlap substantially, suggesting no statistically meaningful sex difference. It would be worthwhile to formally test for sex differences by including a sex-by-exposure interaction term in the regression models. If no statistical evidence for interaction is found, presenting combined estimates for males and females may be more appropriate.

The reviewer is referring to Figure 4, which presents hazard ratios (HRs) for unnatural death comparing individuals with and without a history of non-fatal self-harm. We fitted separate models for each sex. In age-adjusted models, females with a prior non-fatal self-harm encounter had an almost fivefold higher risk of unnatural death (HR 4.63, 95% CI 3.00–7.15) compared to females without such history. In males, prior non-fatal self-harm was associated with a sevenfold higher risk (HR 7.03, 95% CI 5.27–9.39). The same pattern was observed in fully adjusted models and in analyses by method of self-harm (highly lethal, less lethal vs. no prior self-harm). In all analyses, the confidence intervals overlapped between sexes. Importantly, overlapping confidence intervals from separate models cannot be interpreted as evidence of an absence of effect heterogeneity.

Our decision to present sex-specific models was based on pronounced differences in the epidemiology of suicidal behaviors between sexes in South Africa. In our study, females had double the incidence of self-harm (183 per 100,000 person-years; 95% CI 178–188) compared with males (90 per 100,000 person-years; 95% CI 87–94; Table A in Appendix S1 and Figure 1), while males have a much higher risk of unnatural death after non-fatal self-harm: the five-year cumulative incidence of unnatural death after non-fatal self-harm was more than threefold higher in males than in females (Figure 3), with non-overlapping confidence intervals. More broadly, in South Africa, suicide rates in males are more than three times higher than in females [6], although females have twice the lifetime prevalence of suicide attempts [7]. We therefore considered it important to report sex-specific associations, regardless of statistical evidence for interaction.

Nonetheless, we agree that including a combined model and a formal interaction test adds value. We have now included an age- and sex-adjusted combined model. In this model, non-fatal self-harm was associated with a sixfold increase in the risk of unnatural death (HR 6.01, 95% CI 4.73–7.65). The formal test for interaction was marginally significant (p = 0.079), which is not unexpected given the limited number of events (n = 68). We have added this analysis to the Results section and described it in the Methods. While the combined model provides an overall estimate, we believe the sex-specific associations are more informative given the pronounced sex differences in the epidemiology of suicidal behaviors.

We describe the methods for this additional analysis in lines 201-205:

“Third, we fitted a combined Cox model including both males and females, using age as the time scale and adjusting for sex to estimate association between prior non-fatal self-harm and unnatural death across sexes. In addition, we fitted the same model with an interaction term between sex and prior non-fatal self-harm to formally test for effect modification.”

The corresponding results are reported in lines 270-272:

“In a combined model including both sexes and adjusting for age and sex, non-fatal self-harm was associated with a sixfold higher risk of unnatural death (HR 6.01, 95% CI 4.73–7.65). The interaction between sex and prior self-harm showed weak statistical evidence for effect modification (p = 0.079).”

Reviewer #3: I thank the authors for their careful and thorough attention to the reviewer comments. My comments have now been adequately addressed.

Thank you for your positive feedback. We appreciate your comments and are glad that our revisions have addressed your concerns.

--------------------------------------------------------- ---
General editorial requests:

*       We ask every co-author listed on the manuscript to fill in a contributing author statement, making sure to declare all competing interests. If any of the co-authors have not filled in the statement, we will remind them to do so when the paper is revised. If all statements are not completed in a timely fashion this could hold up the re-review process. If new competing interests are declared later in the revision process, this may also hold up the submission. Should there be a problem getting one of your co-authors to fill in a statement we will be in contact. Please do not add or remove authors without first discussing this with the handling editor. You can see our competing interests policy here: <https://journals.plos.org/plosmedicine/s/competing-interests>

All authors declare no competing interests. Please let us know if we need to complete any additional forms and kindly share them with us if so.

* Financial disclosure: Thank you for including your financial enclosure. Please also include URLs from the funders in this statement.

## We included the URLs as requested:

**“Role of the funding source**

Research reported in this publication was supported by the Swiss National Science Foundation (grant numbers 193381, and 189498; <https://www.snf.ch/en>) and the US National Institutes of Health (the National Institute of Allergy and Infectious Diseases, the Eunice Kennedy Shriver National Institute of Child Health and Human Development, the National Cancer Institute, the National Institute of Mental Health, the National Institute on Drug Abuse, the National Heart, Lung, and Blood Institute, the National Institute on Alcohol Abuse and Alcoholism, the National Institute of Diabetes and Digestive and Kidney Diseases, and the Fogarty International Center) (grant number U01AI069924; <https://www.nih.gov>). Study funders had no role in study design; data collection, analysis, or interpretation; or writing the manuscript.”

*  Ethics statement: Could you please include IRB approval numbers?

## We included the IRB approval numbers as requested at the end of the first paragraph of the Methods section:

“The Human Research Ethics Committee of the University of Cape Town (HREC REF Number: 84/2006) and the Cantonal Ethics Committee of the Canton of Bern granted (KEK-Nr: 150/14) permission to analyze data.”

FORMATTING - GENERAL

*   Thank you for including an Author summary.
-Can you please limit the number of bullet points under 'What Did The Researchers Do And Find' to 3?
-Can you please include limitations in the final bullet point of 'What Do These Findings Mean?

## We have limited the 'What Did the Researchers Do and Find?' section to three bullet points and included study limitations in the final bullet point of 'What Do These Findings Mean?'.

### “Why Was This Study Done?

- People who have harmed themselves in the past are more likely to do so again and are at higher risk of dying by suicide or other unnatural means.
- With the right support from health care providers, self-harm can often be prevented.
- Although self-harm is common in African countries, there is limited information to guide how health services can best support people affected by it.

What Did the Researchers Do and Find?

- In a cohort of over 1.35 million members of South African medical insurance schemes, each year, around 2 in every 1,000 females and 1 in every 1,000 males accessed healthcare following self-harm.
- The risk of self-harm is especially high among adolescents and young adults aged 15–24, and among people with mental health or substance use problems.
- Individuals with a history of self-harm were at much greater risk of dying from unnatural causes, such as suicide, in the years following the self-harm event. The risk of dying from unnatural causes was particularly high among men.

What Do These Findings Mean?

- South Africa is facing a high burden of self-harm. Young adults, females, and individuals with mental health or substance use conditions are at increased risk and represent key populations for targeted screening and early intervention.
- Individuals presenting for self-harm, especially males, are at high risk of dying from unnatural causes and may benefit from suicide-specific brief interventions, such as safety planning, motivational techniques, and structured follow-up care.
- The main limitations of the study are that we may have missed some cases of self-harm because we relied on routine medical records, and that we could not examine suicide mortality because the exact cause of death was not available.”

ADDIDTIONAL CHANGES

During an independent code and data review by the senior author, we identified two errors: (1) an error in the time window used to define mental disorders before the self-harm encounter, and (2) an error in the censoring strategy in the mortality analysis for members of the HIV disease management program. These errors had no impact on the conclusions of the manuscript. The changes made to address them and their implications for the results are described in detail below.

1) **Time window to define mental disorders before the self-harm encounter**

We noted an error in Table 2. The estimates for “Mental health diagnoses before the self-harm encounter” and, as a consequence, “New mental health diagnoses within 7 days of the self-harm encounter” were incorrect due to a coding error in the script used to generate the table. The prevalence of mental health diagnoses before the self-harm encounter had incorrectly included diagnoses made on the day of the encounter. We have corrected this error: the prevalence before the self-harm encounter decreased from 62.8% to 50.4%, and the prevalence of new mental health diagnoses within 7 days increased from 14.0% to 23.7%. We have updated Table 2 and revised the relevant text in the manuscript (second paragraph of the Results and third paragraph of the Discussion). All changes are highlighted in yellow.

## Results

“Among 7,510 individuals (0.6%) with a self-harm encounter (5,208 females, 2,302 males), most were hospital admissions (80%). Less lethal methods, mainly poisoning, accounted for 97.8% of cases; highly lethal methods were rare (2.2%). Median age at self-harm encounter was 31 years (IQR 20-40). Half of the cases (50.4%) had received a mental health diagnosis before the self-harm encounter, with another 23.7% being diagnosed within 7 days after the encounter (Table 2).”

## Discussion

“Mental disorders were strong predictors of self-harm. Half of the self-harm cases in our study had a pre-existing mental health diagnosis, and 24% were newly diagnosed within 7 days post-incident, suggesting previously unrecognized illness. The psychiatric morbidity observed in this study is slightly higher than in other LMICs, where fewer than half of those engaging in suicidal behavior had a psychiatric disorder [40]. In contrast, in Western countries, 90% of hospital-presenting self-harm cases had a mental disorder [41]. Growing evidence from LMICs highlights the role of socioeconomic and psychosocial stressors in self-harm [21,22,42], indicating that prevention strategies must address both mental illness and psychosocial factors [38,43,44].”

We corrected an additional inconsistency in the manuscript text: the upper limit of the time window for new mental health diagnoses after self-harm should be 7 days, not 30 days. This has been corrected in the manuscript.

2) **Censoring strategy in the mortality analysis for members of the HIV disease management program**

We identified an error in the censoring strategy used in the mortality analysis for members of the HIV disease management program. In the previous version of the analysis, we followed program members from baseline until the earliest of end of insurance coverage, death, or administrative censoring at HIV program database closure (1 Oct 2022). Because the vital registration database closure date (21 Jan 2021) preceded the HIV program database closure date, program members were no longer at risk of unnatural death after 21 Jan 2021 and should have been administratively censored at that date. The larger cohort of medical scheme beneficiaries was not affected by this issue because the medical scheme database closure date (30 Jun 2020) preceded the vital registration database closure date (21 Jan 2021), and our previous censoring at the medical scheme database closure date was correct in this instance.

We have corrected the censoring strategy for the HIV program members, rerun all mortality analyses, and updated the corresponding tables and figures. Revised estimates in the text and updated tables and figures, are highlighted in green. The changes are described in detail below.

A total of 65 individuals were excluded from the mortality analysis because they joined the HIV program after vital registration database closure. We have revised the Study inclusion flowchart (Figure A in Appendix S1) and lines 250-254 in the Results section accordingly.

The results from the mortality analysis changed minimally. The cumulative incidence of unnatural death and hazard ratios for the associations between non-fatal self-harm and unnatural death, as well as between self-harm method and unnatural death, changed at the second decimal place.

For example, in males who survived a self-harm event, the five-year cumulative incidence of unnatural death changed from 3.41% (95% CI 2.37–4.75) to 3.43% (95% CI 2.38–4.76). In female, the estimate remained unchanged. The hazard ratio for non-fatal self-harm as a predictor of unnatural death changed from 7.02 (95% CI 5.26–9.37) to 7.03 95% CI 5.27–9.39 in males and from 4.61 (95% CI 2.99–7.12) to 4.63 (95% CI 3.00–7.15) in females.

REFERENCES

1.	Hemingway H, Croft P, Perel P, Hayden JA, Abrams K, Timmis A, et al. Prognosis research strategy (PROGRESS) 1: A framework for researching clinical outcomes. BMJ. 2013;346: e5595–e5595. doi:10.1136/bmj.e5595

2.	Riley RD, Hayden JA, Steyerberg EW, Moons KGM, Abrams K, Kyzas PA, et al. Prognosis Research Strategy (PROGRESS) 2: Prognostic Factor Research. PLoS Med. 2013;10: e1001380. doi:10.1371/journal.pmed.1001380

3.	Steyerberg EW, Moons KGM, van der Windt DA, Hayden JA, Perel P, Schroter S, et al. Prognosis Research Strategy (PROGRESS) 3: Prognostic Model Research. PLoS Med. 2013;10: e1001381. doi:10.1371/journal.pmed.1001381

4.	Collins GS, Reitsma JB, Altman DG, Moons K. Transparent reporting of a multivariable prediction model for individual prognosis or diagnosis (TRIPOD): the TRIPOD Statement. BMC Med. 2015;13: 1. doi:10.1186/s12916-014-0241-z

5.	McShane LM, Altman DG, Sauerbrei W, Taube SE, Gion M, Clark GM. REporting recommendations for tumour MARKer prognostic studies (REMARK). Br J Cancer. 2005;93: 387–391. doi:10.1038/sj.bjc.6602678

6.	World Health Organization. Suicide Rates. 2025 [cited 4 Apr 2025]. Available: thtps://www.who.int/data/gho/data/themes/mental-health/suicide-rates

7.	Joe S, Stein DJ, Seedat S, Herman A, Williams DR. Non-fatal suicidal behavior among South Africans. Soc Psychiatry Psychiatr Epidemiol. 2008;43: 454–461. doi:10.1007/s00127-008-0348-7
