# Supplementary material for: Incidence and prognostic factors of self-harm and subsequent unnatural death in South Africa: A cohort study
Source: PLoS Med. 2025 Sep 26;22(9):e1004765. doi: 10.1371/journal.pmed.1004765 (PMC12503312; doi:10.1371/journal.pmed.1004765)
Supplement: Attachment — Submitted filename: Responses_to_reviewers_v1.0.docx [file pmed.1004765.s008.docx]

Response to Editors’ and Reviewers’ Comments

Dear Dr De Bruijn, dear Editors,

We thank the editors and reviewers for their thoughtful evaluation of our revised manuscript (PMEDICINE-D-25-00406R3). We appreciate the opportunity to address the remaining editorial points. Revisions to the manuscript text are highlighted in yellow. Minor formatting changes to the presentation of statistical information and definitions were not highlighted. Below, we provide a point-by-point response to each editorial comment. Our responses are shown in blue.

Sincerely,

Andreas Haas (on behalf of all co-authors)

GENERAL EDITORIAL REQUESTS
* Please change your title to: "Self-harm incidence rates and their predictive value for subsequent unnatural death in South Africa: a Cohort Study"

We are grateful for the editor’s proposal for a more descriptive title. However, the suggested wording could be understood as indicating that we used incidence rates as predictor of unnatural death, which was not the case. To avoid this potential ambiguity, we propose the following alternative:

“Incidence and Prognostic Factors of Self-Harm and Subsequent Unnatural Death in South Africa: A Cohort Study”

* Please ensure that all abbreviations are defined at first use throughout the text.

We confirm that all abbreviations are defined at first use throughout the text.

* Please confirm that all numbers presented in the abstract are present and identical to numbers presented in the main manuscript text.

We confirm that all numbers presented in the abstract are present in the main manuscript text and are identical.

GENERAL
* Statistical reporting: Please revise throughout the manuscript, including tables and figures.
- Please report statistical information as follows to improve clarity for the reader ""22% (95% CI [13,28]; p</=)"".

We confirm that we revised the statistical reporting throughout the manuscript, including tables and figures, to follow the requested format.

- Please separate upper and lower bounds with commas instead of hyphens as the latter can be confused with reporting of negative values.

We replaced the hyphens between the upper and lower bounds of the confidence intervals with commas to avoid confusion with negative values.

- Please repeat statistical definitions (HR, CI etc.) for each set of parentheses.

We repeat statistical definitions for each set of parentheses as requested.

* Please consider moving some of the methods from the supplements into the main text.

We moved the following paragraph from the methods in the supplement to the main text:

“Finally, we used descriptive statistics to compare healthcare utilization prior to self-harm encounters with that of matched controls prior to hypothetical encounter dates. For this analysis, each self-harm case was matched to two randomly selected controls who had no self-harm encounter. Matching criteria included the same baseline year, age group (10-14, 15-19, 20-39, 40-59, 60+), and duration of follow-up (<2 years, 2-4 years, 4-6 years, 6-8 years, or 8+ years). The hypothetical event date for controls was set as their baseline date plus the interval from baseline to the self-harm encounter of the matched case. We calculated standardized differences to assess groups group differences, interpreting values <0.1 as not meaningful, >0.2 as moderate, and >0.5 as large [37].”

*ABSTRACT: please mention clearly that 2011-2022 were the years for which you have data.

The study period is clearly stated in lines 8-9 of the abstract:

“We conducted a cohort study using insurance claims and vital registration data from beneficiaries of South African medical insurance schemes (2011-2022), aged 10 years or older.”

FUNDING STATEMENT
* Thank you for including your funding statement. Could you please include the initials of the people who obtained the grant?

We added the initials of the people who obtained the grants as requested:

“Research reported in this publication was supported by the Swiss National Science Foundation (grant numbers 193381 [AH], and 189498 [ME]; https://www.snf.ch/en) and the US National Institutes of Health (the National Institute of Allergy and Infectious Diseases, the Eunice Kennedy Shriver National Institute of Child Health and Human Development, the National Cancer Institute, the National Institute of Mental Health, the National Institute on Drug Abuse, the National Heart, Lung, and Blood Institute, the National Institute on Alcohol Abuse and Alcoholism, the National Institute of Diabetes and Digestive and Kidney Diseases, and the Fogarty International Center) (grant number U01AI069924 [MD]; https://www.nih.gov). The funders had no role in study design, data collection and analysis, decision to publish, or preparation of the manuscript.”

DATA AVAILABILITY
* Thank you for providing a github/Zenodo link in the data availability statement in the manuscript. Can you please ensure the same statement, including the code availability, is also present in the metadata.

We confirm that the data availability statement in the metadata is identical to the statement in the manuscript.

FIGURES
* Please consider if moving some of the figures shared in the supplementary information into the main text would aid the reader. Specifically, we would suggest to move Figure A from the supplements to the main text.

We have moved Figure A from the supplementary information to the main text, as suggested.

OBSERVATIONAL, COHORT, CROSS-SECTIONAL, AND CASE CONTROL STUDIES

*Thank you for including the STROBE checklist, and mentioning this in your methods section. Could you please spell out STROBE? "This study is reported as per the Strengthening the Reporting of Observational Studies in Epidemiology (STROBE) guideline (Appendix S4)."

We have spelled out STROBE in the Methods section as suggested.

-When completing the checklist, please use section and paragraph numbers, rather than page numbers.

We have revised the STROBE checklist and now indicate the section and subheading, or the section and paragraph number instead of the page numbers.
